# Bosutinib versus imatinib for newly diagnosed chronic phase chronic myeloid leukemia: final results from the BFORE trial

**DOI:** 10.1038/s41375-022-01589-y

**Published:** 2022-05-28

**Authors:** Tim H. Brümmendorf, Jorge E. Cortes, Dragana Milojkovic, Carlo Gambacorti-Passerini, Richard E. Clark, Philipp le Coutre, Valentin Garcia-Gutierrez, Charles Chuah, Vamsi Kota, Jeffrey H. Lipton, Philippe Rousselot, Michael J. Mauro, Andreas Hochhaus, Rafael Hurtado Monroy, Eric Leip, Simon Purcell, Anne Yver, Andrea Viqueira, Michael W. Deininger, BFORE study investigators

**Affiliations:** 1 Universitätsklinikum RWTH Aachen, Aachen, Germany; 2 Georgia Cancer Center, Augusta, GA USA; 3 Hammersmith Hospital, London, UK; 4 University of Milano-Bicocca, Monza, Italy; 5 Department of Molecular & Clinical Cancer Medicine, University of Liverpool, Liverpool, UK; 6 Charité Universitätsmedizin Berlin, Berlin, Germany; 7 Hospital Universitario Ramón y Cajal, Ramón y Cajal Health Research Institute, Madrid, Spain; 8 Singapore General Hospital, Duke-NUS Medical School, Singapore, Singapore; 9 Princess Margaret Cancer Centre, Toronto, ON Canada; 10 Centre Hospitalier de Versailles, Le Chesnay, France; 11 Memorial Sloan Kettering Cancer Center, New York, NY USA; 12 Klinik für Innere Medizin II, Universitätsklinikum Jena, Jena, Germany; 13 Hospital Angeles Pedregal, Mexico City, Mexico; 14 Pfizer Inc, Cambridge, MA USA; 15 Pfizer Ltd, London, UK; 16 Pfizer Inc, Paris, France; 17 Pfizer SLU, Madrid, Spain; 18 University of Utah Health Care, Salt Lake City, UT USA

**Keywords:** Randomized controlled trials, Haematological cancer

## Abstract

This analysis from the multicenter, open-label, phase 3 BFORE trial reports efficacy and safety of bosutinib in patients with newly diagnosed chronic phase (CP) chronic myeloid leukemia (CML) after five years’ follow-up. Patients were randomized to 400-mg once-daily bosutinib (*n* = 268) or imatinib (*n* = 268; three untreated). At study completion, 59.7% of bosutinib- and 58.1% of imatinib-treated patients remained on study treatment. Median duration of treatment and time on study was 55 months in both groups. Cumulative major molecular response (MMR) rate by 5 years was higher with bosutinib versus imatinib (73.9% vs. 64.6%; odds ratio, 1.57 [95% CI, 1.08–2.28]), as were cumulative MR^4^ (58.2% vs. 48.1%; 1.50 [1.07–2.12]) and MR^4.5^ (47.4% vs. 36.6%; 1.57 [1.11–2.22]) rates. Superior MR with bosutinib versus imatinib was consistent across Sokal risk groups, with greatest benefit seen in patients with high risk. Treatment-emergent adverse events (TEAEs) were consistent with 12-month data. After 5 years of follow-up there was an increase in the incidence of cardiac, effusion, renal, and vascular TEAEs in bosutinib- and imatinib-treated patients, but overall, no new safety signals were identified. These final results support 400-mg once-daily bosutinib as standard-of-care in patients with newly diagnosed CP CML.

This trial was registered at www.clinicaltrials.gov as #NCT02130557.

## Introduction

Bosutinib is approved for the treatment of patients with Philadelphia chromosome–positive (Ph+) chronic myeloid leukemia (CML) resistant/intolerant to prior therapy and patients with newly diagnosed chronic phase (CP) CML [1–6]. Approval of first-line bosutinib was based on primary results from the phase 3 BFORE trial, which showed superior efficacy of bosutinib versus imatinib in the modified intent-to-treat (mITT) population (Ph+ patients with e13a2/e14a2 transcripts) after ≥12 months of follow-up [7]. We report the final efficacy and safety results from BFORE after five years of follow-up.

## Methods

### Study design and patients

BFORE (ClincalTrials.gov, NCT02130557) was an open-label, randomized, multicenter, phase 3 study; methods have been published [7, 8]. Patients aged ≥18 years, with newly diagnosed *BCR::ABL1*-positive CP CML, were randomized 1:1 to receive (starting dose) bosutinib or imatinib 400 mg once daily. On-study treatment was continued for five years (240 weeks; end of study) or until treatment failure, unacceptable toxicity, death, or withdrawal of consent. Patients who discontinued treatment prior to completing five years were followed for survival until completion of five years on study, death, or withdrawal of consent. At the end of the planned five years, patients could continue with their ongoing treatment at the discretion of the investigator.

The primary endpoint was major molecular response (MMR; *BCR::ABL1* ≤ 0.1% on the international scale [IS]) at 12 months (mITT population).

The study was conducted in accordance with the Declaration of Helsinki. Patients provided written informed consent, and the protocol was approved by study-site institutional review boards. This final analysis was based on a last patient/last visit of 17 April 2020 (12 June 2020 database lock), five years after the last patient enrolled.

### Efficacy and safety assessments

The short-term secondary endpoint, MMR by month 18 (not previously reported), is included. Long-term secondary endpoints included duration of complete cytogenetic response (CCyR), duration of MMR, on-treatment event-free survival (EFS), and overall survival (OS). Exploratory endpoints included time to response (TTR), on-treatment transformation to accelerated phase (AP) or blast phase (BP) CML, and newly observed *BCR::ABL1* mutations. Post-hoc analyses included cumulative response rates by five years, cumulative molecular response (MR) rate by Sokal risk group, duration of MR^4^, sustained 1-year MR^4^ (≥3 years on treatment, and *BCR::ABL1* ≤ 0.01% IS in all consecutive assessments for ≥1 year), sustained two-year MR^4^ (≥4 years on treatment, and *BCR::ABL1* ≤ 0.01% IS in all consecutive assessments for ≥2 years), and efficacy by *BCR::ABL1* ≤ 10% IS at three months in evaluable patients with ≥3000 ABL1 copies at three months. Additional methods are provided in the supplementary material.

### Statistical analysis

This analysis evaluated efficacy in the ITT population (all randomized patients), with the exception of cytogenetic endpoints, which were evaluated in the mITT population (Ph+ patients with e13a2 and/or e14a2 transcripts). Results for prespecified endpoints in the hierarchical testing strategy are displayed for the ITT population (results were consistent with those in the mITT population [data not shown]). Per protocol, CCyR was imputed on any date where MMR was achieved and no valid cytogenetic assessment was available. Definitions for TTR, duration of response, on-treatment EFS and OS, censoring for time-to-event endpoints, and imputation methods have been described [7].

All efficacy analyses were based on assessments up through 28 days after the last dose of study medication except for OS, which included posttreatment follow-up data. Response data after treatment discontinuation were not collected.

Confirmed loss of response was defined as two consecutive assessments at least 28 days apart, treatment discontinuation due to suboptimal response/treatment failure or progressive disease, or death due to progressive disease within 28 days of last dose. Confirmed loss of *BCR::ABL1* transcripts ≤1% IS was included as an additional EFS event for Philadelphia chromosome–negative/unknown Philadelphia chromosome status e13a2/e14a2 patients. Duration of response was measured from the first date of response until the first date of loss of response that was subsequently confirmed. Loss of CCyR was defined as ≥1 Ph+ metaphase from <100 metaphases analyzed. Loss of MMR and MR^4^ was defined as *BCR::ABL1* transcripts >0.1% and >0.01% IS, respectively, with ≥5-fold increase from the lowest recorded value.

Safety data were summarized descriptively and included all randomized patients who received ≥1 dose of study medication.

All hazard or odds ratios are bosutinib vs imatinib. Ratios <1 for duration of response, EFS, and OS, and ratios >1 for response and TTR were considered to favor bosutinib. For all endpoints, 95% confidence intervals (CIs) excluding 1 were considered predictive of the outcome of interest.

## Results

### Disposition, demography, and baseline characteristics

**Fig. 1:**
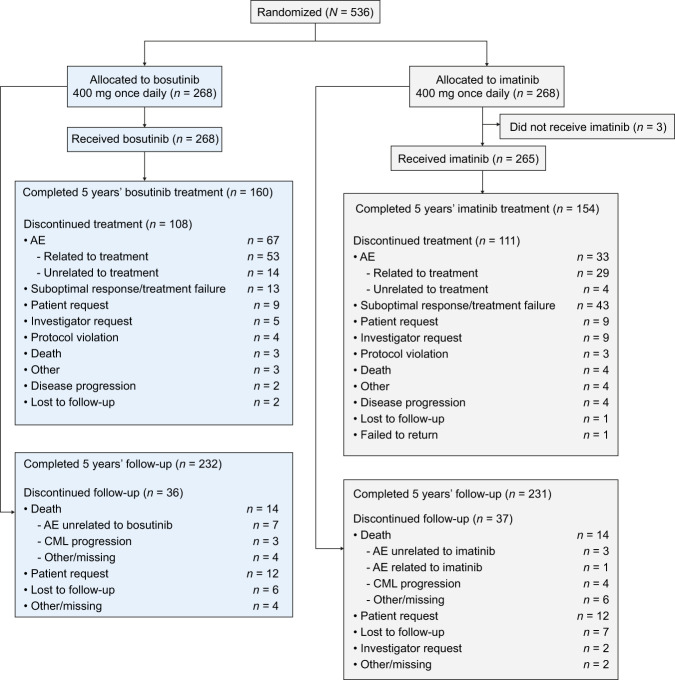
Patient disposition. *AE* adverse event.

A total of 536 patients were randomized to bosutinib (*n* = 268) or imatinib (*n* = 268), of whom 268 and 265, respectively, received study treatment (Fig. 1). Patient baseline demographics and disease characteristics (ITT population) were well balanced across treatment arms (Supplementary Table 1). The median age at study entry was 53 years (range, 18–84) and 58% were male. Most patients were Ph+ (92%) and had typical *BCR::ABL1* transcripts (98.5%). In the bosutinib- versus imatinib-treated patients, 57.8% versus 56.2% of patients had ≥1 cardiovascular risk factor at baseline; 20.9% versus 17.7% had ≥3 risk factors. Baseline risk factors are shown in Supplementary Table 2.

Median duration of treatment and time on study was 55 months for bosutinib and imatinib patients (Table 1); respective median (range) dose intensity was 393.6 (39–583) versus 400.0 (189–765) mg/d. At study completion, 59.7% versus 58.1% of bosutinib- versus imatinib-treated patients were still receiving treatment; 86.6% versus 86.2% of randomized patients completed five years of follow-up. The most common primary reasons for permanent treatment discontinuation were adverse events (AEs) in the bosutinib arm (bosutinib, 25.0% vs. imatinib, 12.5%) and lack of efficacy (suboptimal response, treatment failure, or disease progression) in the imatinib arm (imatinib, 17.7% vs. bosutinib, 5.6%; Fig. 1).

**Table 1:** Duration of treatment and cumulative MR rates by 60 months.

	Bosutinib	Imatinib	
Duration of treatment, mo	*n* = 268	*n* = 265	
Median (range)	55.1 (0.3–60.1)	55.0 (0.7–56.8)	
Cumulative response rates, % (95% CI)	*n* = 268	*n* = 268	OR^a^ (95% CI)
MMR	73.9 (68.6–79.1)	64.6 (58.8–70.3)	1.57 (1.08–2.28)
MR^4^	58.2 (52.3–64.1)	48.1 (42.2–54.1)	1.50 (1.07–2.12)
MR^4.5^	47.4 (41.4–53.4)	36.6 (30.8–42.3)	1.57 (1.11–2.22)
Cumulative molecular response rates by Sokal risk group at screening, % (95% CI)			
Low risk	*n* = 95	*n* = 106	OR^a^ (95% CI)
MMR	75.8 (67.2–84.4)	72.6 (64.2–81.1)	1.18 (0.63–2.22)
MR^4^	60.0 (50.1–69.9)	55.7 (46.2–65.1)	1.20 (0.68–2.10)
MR^4.5^	53.7 (43.7–63.7)	42.5 (33.0–51.9)	1.57 (0.90–2.74)
Intermediate risk	*n* = 117	*n* = 105	
MMR	74.4 (66.4–82.3)	63.8 (54.6–73.0)	1.65 (0.93–2.92)
MR^4^	56.4 (47.4–65.4)	46.7 (37.1–56.2)	1.48 (0.87–2.51)
MR^4.5^	42.7 (33.8–51.7)	37.1 (27.9–46.4)	1.26 (0.74–2.17)
High risk	*n* = 56	*n* = 57	
MMR	69.6 (57.6–81.7)	50.9 (37.9–63.9)	2.22 (1.03–4.79)
MR^4^	58.9 (46.0–71.8)	36.8 (24.3–49.4)	2.46 (1.15–5.24)
MR^4.5^	46.4 (33.4–59.5)	24.6 (13.4–35.7)	2.66 (1.20–5.92)

All ratios are bosutinib vs. imatinib. OR > 1 favor bosutinib.

*CI* confidence interval, *MMR* major molecular response, *MR* molecular response, *OR* odds ratio.

^a^Overall adjusted for Sokal risk group and region as determined at the time of randomization and unadjusted for subgroups.

More patients receiving bosutinib versus imatinib had dose interruptions (68.7% vs. 45.7%) or dose reductions (45.5% vs. 24.5%); fewer patients required dose escalations to >400 mg once daily (21.6% vs. 31.3%).

### Efficacy

**Fig. 2:**
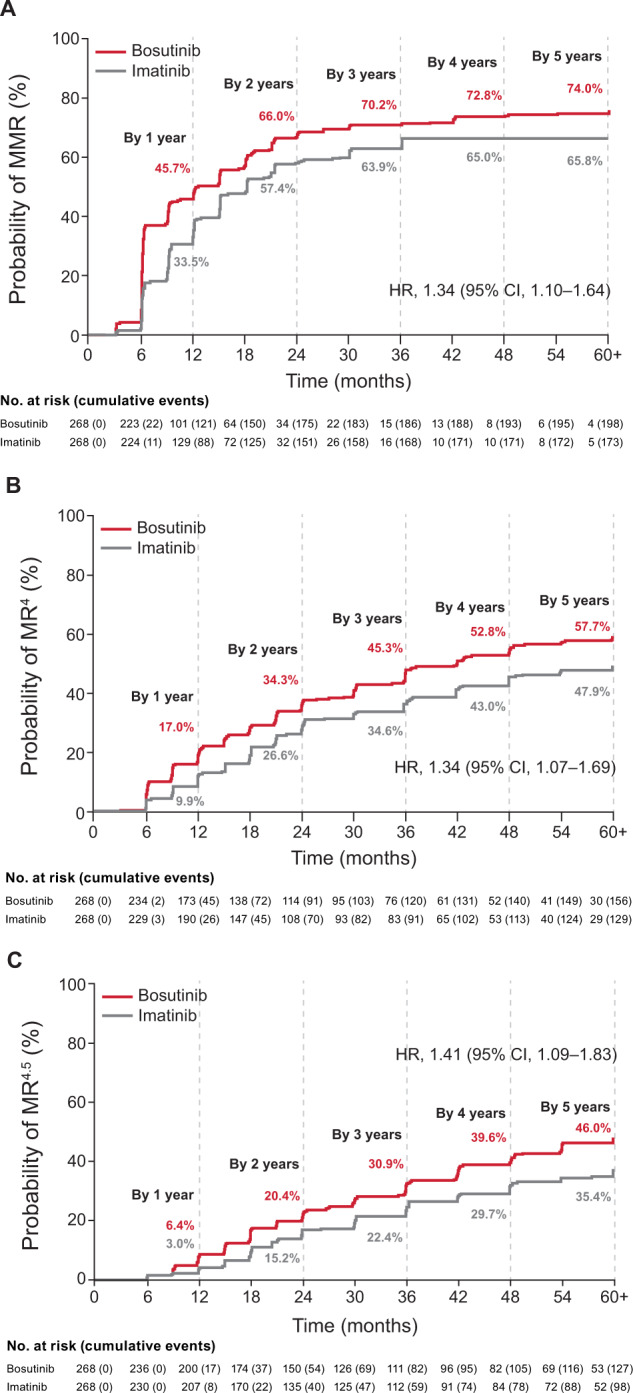
Cumulative incidence of molecular response. **A** MMR. **B** MR^4^. **C** MR^4.5^. CI confidence interval, HR hazard ratio, MMR major molecular response, MR molecular response.

The primary endpoint, MMR at 12 months, and secondary endpoint, CCyR by month 12, were significantly higher for bosutinib versus imatinib [7]. MMR by 18 months (secondary endpoint) was not statistically significantly higher with bosutinib versus imatinib at the prespecified 1-sided 0.0125 level (60.8% vs. 51.5%; OR, 1.47 [95% CI, 1.04–2.08], 1-sided *P* = 0.014). At the final analysis, the cumulative MMR rate by 60 months was higher with bosutinib versus imatinib (73.9% vs. 64.6%; OR, 1.57 [95% CI, 1.08–2.28]), as were the cumulative rates for MR^4^ (58.2% vs. 48.1%; OR, 1.50 [95% CI, 1.07–2.12]) and MR^4.5^ (47.4% vs. 36.6%; OR, 1.57 [95% CI, 1.11–2.22]) (Table 1). Patients receiving bosutinib achieved responses earlier compared with imatinib; the cumulative incidence function for MMR, MR^4^, and MR^4.5^ was higher with bosutinib (Fig. 2). Superior MR with bosutinib versus imatinib was observed across Sokal risk groups, with the greatest difference between treatment arms in patients with Sokal high-risk (Table 1).

The cumulative CCyR rate by 60 months (mITT population) was similar for patients receiving bosutinib versus imatinib (83.3% vs. 76.8%, OR, 1.52 [95% CI, 0.97–2.39]), but the cumulative incidence function of CCyR was higher with bosutinib (hazard ratio [HR], 1.35 [95% CI, 1.11–1.64]).

Among responders, there were no differences between treatment arms in the duration of MMR (HR, 1.01 [95% CI, 0.46–2.23]) and MR^4^ (HR, 0.99 [95% CI, 0.43–2.25]). At 4 years, the probability (95% CI) of maintaining MMR was 92.6% (87.6–95.7) with bosutinib versus 91.8% (85.9–95.3) with imatinib; the probability of maintaining MR^4^ was 89.7% (82.1–94.1) versus 88.8% (80.3–93.7). Of responders, there were 13 (6.6%) bosutinib- and 12 (6.9%) imatinib-treated patients with confirmed loss of MMR; five and two patients, respectively, subsequently regained MMR with continued treatment. Similarly, of 12 (7.7%) and 11 (8.5%) patients, respectively, with confirmed loss of MR^4^, four patients in the bosutinib arm subsequently regained MR^4^.

The duration of CCyR was similar with bosutinib and imatinib responders (HR, 0.39 [95% CI, 0.14–1.13]); the probability (95% CI) of maintaining CCyR at four years was 97.4% (93.9–98.9) and 93.7% (88.9–96.5), respectively.

The rate of patients achieving a sustained MR^4^ was also assessed. In the bosutinib versus imatinib arms, 42.9% (95% CI, 37.0–48.8) versus 36.2% (95% CI, 30.4–41.9) (OR, 1.32 [95% CI, 0.94–1.87]) of patients had a one-year sustained MR^4^, and 32.5% (95% CI, 26.9–38.1) versus 26.5% (95% CI, 21.2–31.8) (OR, 1.33 [95% CI, 0.92–1.93]) of patients had a two-year sustained MR^4^. In a subdistributional hazards model, *BCR::ABL1* transcript level ≤10% at three months was predictive of time to a one-year sustained MR^4^, and Eastern Cooperative Oncology Group performance status 0 and *BCR::ABL1* transcript level ≤10% at three months were predictive of time to a two-year sustained MR^4^ (Supplementary Table 3).

On-treatment transformations to AP/BP CML occurred in six bosutinib- and seven imatinib-treated patients. Of these, six (three in each arm) met AP criteria within two weeks of randomization based solely on increased basophil count and did not appear to be true transformations, as their clinical course was not consistent with AP/BP. None of these six patients discontinued treatment due to progression to AP/BP or death. Of the remaining patients, all three bosutinib-treated patients progressed to BP; three imatinib-treated patients progressed to AP and one to BP. There were no transformations after 24 months.

On-treatment EFS was not statistically significantly different between the two treatment arms at the prespecified 1-sided 0.0125 level (HR, 0.70 [95% CI, 0.38–1.27], 1-sided *P* = 0.122); the cumulative incidence (95% CI) of on-treatment progression/death at 60 months was 6.7% (4.1–10.1) for bosutinib versus 9.3% (6.2–13.2) for imatinib. OS was similar between treatment arms (HR, 0.95 [95% CI, 0.45–1.99]): the 60-month probability (95% CI) was 94.5% (90.8–96.7) for bosutinib versus 94.6% (91.0–96.8) for imatinib; due to the prespecified hierarchical testing strategy, statistical significance of OS was not tested, as EFS difference was not statistically significant. Fourteen bosutinib- and imatinib-treated patients in each arm died during the study period; three and four deaths, respectively, were assessed by the investigator as CML-related (Supplementary Table 4).

**Fig. 3:**
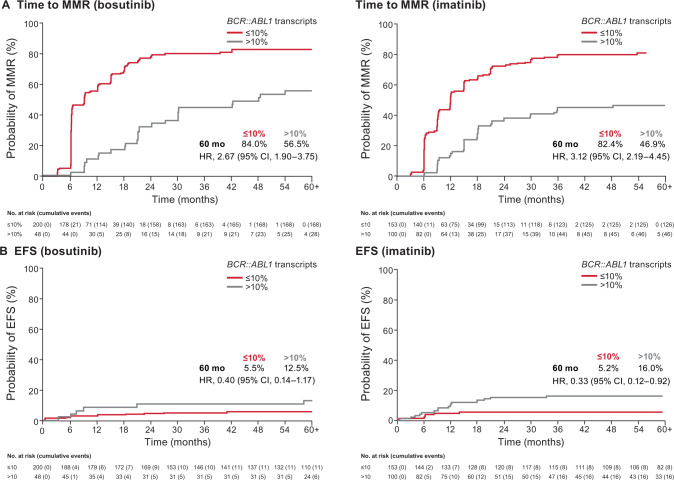
Landmark analysis according to *BCR::ABL1* transcript level (≤10% vs. >10%) at three months. **A** Cumulative incidence of MMR. **B** Cumulative incidence of on-treatment progression/death (EFS). CI confidence interval, EFS event-free survival, HR hazard ratio, MMR major molecular response.

Among evaluable patients, a higher percentage in the bosutinib versus imatinib arm achieved *BCR::ABL1* transcripts ≤10% at three months (80.6% vs. 60.5%; OR, 2.72 [95% CI, 1.82–4.08]), *BCR::ABL1* transcripts ≤1% at three months (38.3% vs. 15.8%; OR, 3.31 [95% CI, 2.16–5.05), and *BCR::ABL1* transcripts ≤1% at six months (75.3% vs. 58.1%; OR, 2.33 [95% CI, 1.55–3.50]). In both treatment arms, the cumulative incidence function of MMR (Fig. 3A), as well as MR^4^ and MR^4.5^ (data not shown), was higher in patients who had *BCR::ABL1* transcripts ≤10% at three months versus those who did not. Cumulative incidence function of on-treatment EFS events by *BCR::ABL1* transcript level (≤10% vs. >10% IS) at three months is shown in Fig. 3B; a lower rate of EFS events was observed for patients with *BCR::ABL1* transcript level ≤10% (vs. >10%) at three months in the imatinib arm.

Overall, 114 (42.5%) and 131 (48.9%) patients in the bosutinib and imatinib arms had *BCR::ABL1* mutation testing at suboptimal response, treatment failure, or at the end of treatment; six (2.2%) and 12 (4.5%) patients, respectively, had detectable mutations: bosutinib: *T3151* (*n* = 5) and *V299L* (*n* = 1); imatinib: *F359V* (*n* = 3), *E459K* (*n* = 2), and *T315I*, *Y253H*, *M244V*, *L248V*, *G250E*, and *E255V* (*n* = 1 each). One imatinib-treated patient had three mutations (*E355G*, *T315I*, and *Y253H*). Overall, most mutations (66.7%) were detected within the first 12 months of treatment (bosutinib, 50.0% and imatinib, 75.0%). In the bosutinib arm, 50.0% (all *T315I*) of mutations were detected in patients who never achieved MMR and 50.0% after treatment failure (after achieving at least MMR). In the imatinib arm, 75.0% of patients with mutations never achieved MMR; two (16.7%) patients developed mutations (*T315I* and *M244V*) after achieving at least MMR, and one (8.3%) patient with an *F359V* mutation initially achieved MMR after detection of the mutation.

### Safety

Any grade treatment-emergent AEs (TEAEs) occurred in 98.9% (grade 3/4: 73.5%) versus 98.9% (grade 3/4: 57.0%) of bosutinib- versus imatinib-treated patients (Supplementary Table 5). Laboratory abnormalities are shown in Supplementary Table 6. Results were similar to those previously reported at the 12-month analysis. Gastrointestinal, liver, and rash TEAEs were more frequent (≥10%) in the bosutinib arm, whereas edema and musculoskeletal TEAEs were more frequent with imatinib (Table 2). The most common newly occurring TEAEs (any grade) after 12 months were increased lipase (9.0%) with bosutinib, and diarrhea (8.3%) with imatinib. In bosutinib- versus imatinib-treated patients, 25.4% versus 14.3% had AEs leading to permanent treatment discontinuation (Supplementary Table 7); 1.5% and 1.1% were due to diarrhea. The majority of discontinuations due to AEs occurred in year 1 (bosutinib, 14.2%; imatinib, 10.6%; Supplementary Fig. 1). Most frequent AEs leading to discontinuation were increased ALT (overall, 4.9%; year 1, 4.5%) with bosutinib versus thrombocytopenia (overall, 1.5%; year 1, 1.5%) with imatinib. AEs leading to bosutinib discontinuation after year 1 in >1% of patients were increased lipase (overall, 1.9%; year 1, 0.7%); no individual AE led to imatinib discontinuation in >1% of patients after year 1. TEAEs resulting in death within 28 days of last dose occurred in three (1.1%) bosutinib- versus four (1.5%) imatinib-treated patients: acute cardiac failure, myocardial ischemia, and renal failure with bosutinib; and pneumonia, sepsis, cerebrovascular accident, and disease progression with imatinib. Only sepsis (in the imatinib arm) was considered related to study drug by the investigator.

**Table 2:** TEAEs of special interest.

	Bosutinib (*n* = 268)	Imatinib (*n* = 265)
TEAE cluster,^a^ *n* (%)	Any grade	Any grade
Any gastrointestinal TEAE	214 (79.9)	163 (61.5)
Diarrhea	201 (75.0)	107 (40.4)
Nausea	100 (37.3)	112 (42.3)
Vomiting	55 (20.5)	54 (20.4)
Any myelosuppression TEAE	128 (47.8)	125 (47.2)
Thrombocytopenia	96 (35.8)	53 (20.0)
Anemia	59 (22.0)	60 (22.6)
Neutropenia	33 (12.3)	61 (23.0)
Leukopenia	18 (6.7)	34 (12.8)
Lymphopenia	15 (5.6)	8 (3.0)
Any liver TEAE	118 (44.0)	41 (15.5)
ALT increased	90 (33.6)	16 (6.0)
AST increased	69 (25.7)	18 (6.8)
Blood bilirubin increased	17 (6.3)	7 (2.6)
Blood alkaline phosphatase increased	17 (6.3)	7 (2.6)
Transaminases increased	8 (3.0)	2 (0.8)
Hyperbilirubinemia	6 (2.2)	1 (0.4)
Any rash TEAE	105 (39.2)	69 (26.0)
Rash	62 (23.1)	39 (14.7)
Rash maculo-papular	14 (5.2)	16 (6.0)
Erythema	13 (4.9)	6 (2.3)
Rash pruritic	10 (3.7)	1 (0.4)
Dermatitis acneiform	9 (3.4)	2 (0.8)
Acne	8 (3.0)	0
Eczema	7 (2.6)	8 (3.0)
Any musculoskeletal TEAE	95 (35.4)	158 (59.6)
Arthralgia	48 (17.9)	49 (18.5)
Back pain	32 (11.9)	25 (9.4)
Pain in extremity	26 (9.7)	39 (14.7)
Myalgia	13 (4.9)	48 (18.1)
Musculoskeletal pain	12 (4.5)	12 (4.5)
Muscle spasms	10 (3.7)	81 (30.6)
Bone pain	8 (3.0)	19 (7.2)
Any edema TEAE	42 (15.7)	115 (43.4)
Edema peripheral	20 (7.5)	43 (16.2)
Weight increased	8 (3.0)	20 (7.5)
Face edema	7 (2.6)	17 (6.4)
Periorbital edema	4 (1.5)	44 (16.6)
Eyelid edema	3 (1.1)	24 (9.1)
Orbital edema	0	6 (2.3)
Any hypertension TEAE	28 (10.4)	29 (10.9)
Hypertension	26 (9.7)	29 (10.9)
Any renal TEAE	28 (10.4)	26 (9.8)
Blood creatinine increased	18 (6.7)	22 (8.3)
Acute kidney injury	6 (2.2)	2 (0.8)
Any cardiac TEAE	26 (9.7)	23 (8.7)
Sinus bradycardia	6 (2.2)	0
Electrocardiogram QT prolonged	4 (1.5)	10 (3.8)
Any metabolic TEAE	24 (9.0)	21 (7.9)
Hypercholesterolemia	13 (4.9)	1 (0.4)
Hyperglycemia	10 (3.7)	16 (6.0)
Any vascular TEAE	20 (7.5)	9 (3.4)
Cardiovascular TEAEs	13 (4.9)	1 (0.4)
Angina pectoris	8 (3.0)	1 (0.4)
Myocardial ischemia	6 (2.2)	0
Cerebrovascular TEAEs	2 (0.7)	3 (1.1)
Peripheral vascular TEAEs	6 (2.2)	6 (2.3)
Any effusion TEAE	16 (6.0)	6 (2.3)
Pleural effusion	14 (5.2)	5 (1.9)

*ALT* alanine aminotransferase, *AST* aspartate aminotransferase, *TEAE* treatment-emergent adverse event.

^a^Investigator-reported TEAEs occurring in >2% of patients at the level of preferred term or in >1% of patients at the level of TEAE cluster in the bosutinib or imatinib arms are reported. Patients may report >1 TEAE within each cluster.

Liver TEAEs were reported in 118 (44.0%) bosutinib- versus 41 (15.5%) imatinib-treated patients; the most common were alanine aminotransferase (ALT) and/or aspartate aminotransferase (AST) increases. Increased ALT and/or AST TEAEs were reported in 91 (34.0%) bosutinib- versus 22 (8.3%) imatinib-treated patients and led to treatment discontinuation in 16 (6.0%) versus no patients.

Cardiac TEAEs were reported in 26 (9.7%) versus 23 (8.7%) patients and led to treatment discontinuation in one bosutinib- (0.4%) versus no imatinib-treated patients. The most common cardiac TEAEs were sinus bradycardia (2.2% vs. 0%) in the bosutinib arm, and electrocardiogram QT prolonged (1.5% vs. 3.8%) in the imatinib arm (Table 2). A medical history of cardiac events was reported by five (19.2%) versus six (26.1%) patients with cardiac TEAEs. Risk factors (HR [95% CI]) for time to initial cardiac TEAEs were a history of cardiac events (3.45 [1.60–7.41]), hypertension TEAEs (3.08 [1.24–7.68]), and vascular TEAEs (5.10 [1.51–17.23]; Supplementary Table 8).

Vascular TEAEs were reported in 20 (7.5%) versus 9 (3.4%) bosutinib- versus imatinib-treated patients, and led to treatment discontinuation in three (1.1%) versus one (0.4%) patient; five (25.0%) versus six (66.7%) patients with vascular TEAEs had a medical history of vascular events. Cardiovascular, cerebrovascular, and peripheral vascular TEAEs, respectively, were reported in 13 (4.9%), two (0.7%), and six (2.2%) patients in the bosutinib arm versus one (0.4%), three (1.1%), and six (2.3%) in the imatinib arm (Table 2). Vascular TEAEs occurring in ≥1% of patients in either treatment arm were angina pectoris (3.0% vs. 0.4%), myocardial ischemia (2.2% vs. 0%), and peripheral coldness (0.4% vs. 1.1%). Risk factors (HR [95% CI]) for time to initial vascular TEAEs were a history of vascular events (4.76 [1.85–12.28]), diabetes (3.05 [1.03–9.07]), and cardiac TEAEs (7.94 [2.37–26.58]). In multivariable analyses, treatment group was not predictive of time (HR [95% CI]) to initial cardiac (0.91 [0.47–1.74]) or vascular (2.23 [0.97–5.09]) TEAEs (Supplementary Table 8). The exposure-adjusted incidence rates (Supplementary Table 9) and cumulative rates per treatment year (Table 3) are presented.

**Table 3:** Cumulative rate of patients with adverse events of special interest, by year.

*n* (%)	Bosutinib (*n* = 268)					Imatinib (*n* = 265)				
	Year 1	Year 2	Year 3	Year 4	Year 5+	Year 1	Year 2	Year 3	Year 4	Year 5+
Cardiac^a^	13 (4.9)	17 (6.3)	20 (7.5)	22 (8.2)	26 (9.7)	11 (4.2)	14 (5.3)	16 (6.0)	19 (7.2)	23 (8.7)
Vascular^b^	9 (3.4)	15 (5.6)	17 (6.3)	19 (7.1)	20 (7.5)	4 (1.5)	4 (1.5)	7 (2.6)	8 (3.0)	9 (3.4)
Cardiovascular	6 (2.2)	10 (3.7)	10 (3.7)	12 (4.5)	13 (4.9)	0	0	1 (0.4)	1 (0.4)	1 (0.4)
Cerebrovascular	0	0	2 (0.7)	2 (0.7)	2 (0.7)	1 (0.4)	1 (0.4)	2 (0.8)	3 (1.1)	3 (1.1)
Peripheral vascular	3 (1.1)	5 (1.9)	5 (1.9)	5 (1.9)	6 (2.2)	3 (1.1)	3 (1.1)	5 (1.9)	5 (1.9)	6 (2.3)
Effusion^c^	6 (2.2)	8 (3.0)	12 (4.5)	16 (6.0)	16 (6.0)	4 (1.5)	4 (1.5)	4 (1.5)	4 (1.5)	6 (2.3)
Pleural effusion	5 (1.9)	7 (2.6)	11 (4.1)	14 (5.2)	14 (5.2)	4 (1.5)	4 (1.5)	4 (1.5)	4 (1.5)	5 (1.9)
Pericardial effusion	1 (0.4)	2 (0.7)	3 (1.1)	4 (1.5)	5 (1.9)	0	0	0	0	1 (0.4)
Renal^d^	16 (6.0)	21 (7.8)	22 (8.2)	26 (9.7)	28 (10.4)	16 (6.0)	22 (8.3)	23 (8.7)	23 (8.7)	26 (9.8)

*CNS* central nervous system, *HLGT* high-level group term, *HLT* high-level term, *MedDRA* Medical Dictionary for Regulatory Activities, *NEC* not elsewhere classified, *PT* preferred term, SMQ standardized MedDRA query, *TEAE* treatment-emergent adverse event.

^a^Includes the MedDRA HLGT: Cardiac arrhythmias, Heart failures; PT: Cardiac death, Sudden cardiac death, Sudden death, Ejection fraction decreased; SMQ: Torsade de pointes/QT prolongation (narrow).

^b^Vascular includes MedDRA terms for cardiovascular, cerebrovascular, and peripheral vascular TEAEs:

• Cardiovascular: HLGT: Coronary artery disorders; HLT: Arterial therapeutic procedures (excluding aortic), Vascular imaging procedures NEC, Vascular therapeutic procedures NEC; PT: Transcatheter arterial chemoembolization.

• Cerebrovascular: HLT: CNS hemorrhages and cerebrovascular accidents, CNS vascular disorders NEC, Transient cerebrovascular events.

• Peripheral vascular: HLGT: Arteriosclerosis, stenosis, vascular insufficiency, and necrosis; Embolism and thrombosis; HLT: Non-site-specific vascular disorders NEC, Peripheral vascular disorders NEC (excluding PTs flushing and hot flush); PT: Intestinal ischemia.

^c^Includes the MedDRA *PT*: Pericardial effusion, Pleural effusion.

^d^Includes the MedDRA HLT: Renal failure and impairment; PT: Blood creatinine abnormal, Blood creatinine increased, Creatinine renal clearance abnormal, Creatinine renal clearance decreased, Glomerular filtration rate abnormal, Glomerular filtration rate decreased.

Effusion TEAEs were reported in 16 (6.0%) bosutinib- versus six (2.3%) imatinib-treated patients and led to treatment discontinuation in two (0.7%) versus no patients. Pleural and pericardial effusions, respectively, occurred in 14 (5.2%) versus five (1.9%) bosutinib- and five (1.9%) versus one (0.4%) imatinib-treated patients. Risk factors (HR [95% CI]) for effusion TEAEs were increasing age (1.08 [1.01–1.16]), no history of tobacco use (<0.001 [<0.001 to <0.001]), history of pulmonary events (3.74 [1.73–8.08]), and treatment with bosutinib (2.98 [1.08−8.19]) (Supplementary Table 8). The exposure-adjusted incidence rates are shown in Supplementary Table 9.

Renal TEAEs were reported in 28 (10.4%) versus 26 (9.8%) patients treated with bosutinib versus imatinib; increased blood creatinine was the most common TEAE in both arms (Table 2). Decreases from baseline in estimated glomerular filtration rate (eGFR) based on the Modification of Diet in Renal Disease method and increases in serum creatinine were observed over time in both treatment arms (Supplementary Fig. 2A, B). At 60 months, median decline from baseline eGFR was 14.1 mL/min/1.73 m^2^ with bosutinib versus 14.6 mL/min/1.73 m^2^ with imatinib; the median increase in blood creatinine was 10.0 μmol/L in the bosutinib arm and 10.1 μmol/L in the imatinib arm. No consistent trend in blood urea nitrogen was observed over time (Supplementary Fig. 2C); median changes from baseline at 60 months were 0.2 mmol/L with bosutinib versus 0.0 mmol/L with imatinib. In the bosutinib and imatinib arms, respectively, 37 (13.8%) and 23 (8.7%) patients had on-treatment eGFR Kidney Disease Improving Global Outcomes grade ≥3b (<45 mL/min/1.73 m^2^); of these, 17 (45.9%) and 12 (52.2%) had an improvement to grade ≤3a at the last recorded assessment. Median blood urea nitrogen values on or after initial eGFR grade ≥3b were above the upper limit of normal in both treatment arms, although they were higher in the bosutinib arm (Supplementary Table 10). Risk factors (HR [95% CI]) for time to initial grade ≥3b eGFR were increasing age (1.06 [1.03–1.08]), race other than White (0.49 [0.26–0.94]), Eastern Cooperative Oncology Group performance status >0 (2.50 [1.49–4.20]), decreased baseline eGFR (0.94 [0.91–0.96]), no history of tobacco use (<0.001 [<0.001 to <0.001]), renal disease (5.74 [2.68–12.30]), and diabetes mellitus (2.78 [1.58–4.89]); Supplementary Table 11); treatment group was not predictive of time to initial grade ≥3b eGFR (1.39 [0.85–2.30]).

## Discussion

This final analysis of the BFORE trial demonstrated long-term efficacy and safety of bosutinib in patients with newly diagnosed CP CML. After 5 years of follow-up, superior MR was demonstrated with bosutinib versus imatinib. An improvement in MR in favor of bosutinib was identified across all Sokal risk groups, with the greatest improvement observed in patients with Sokal high-risk, which is an important factor if treatment-free remission is considered as a treatment goal [9]. Furthermore, the rate of early MR at 3 months was higher with bosutinib than with imatinib and, in both treatment arms, the cumulative incidence of MMR and deep molecular response (DMR; defined as MR^4^ and MR^4.5^) was higher in patients who had *BCR::ABL1* transcripts ≤10% at 3 months versus those who did not.

The 5-year follow-up of the second-generation tyrosine kinase inhibitors (TKIs) nilotinib (ENESTnd trial) and dasatinib (DASISION trial) in patients with newly diagnosed CP CML has been reported [10, 11]. Although comparisons between trials should be considered with caution, MR rates with bosutinib align with the MMR and DMR rates observed with nilotinib and dasatinib. An improvement in cumulative MR rates by 60 months with bosutinib versus imatinib (difference [∆] in response) was demonstrated for bosutinib despite the better-than-expected MR rates in the imatinib arm in BFORE relative to the other two trials (MMR 73.9%, ∆9.3%; MR^4.5^ 47.4%, ∆10.8%), nilotinib (300 mg twice daily: MMR 77.0%, ∆16.6%; MR^4.5^ 53.5%, ∆22.1%), and dasatinib (MMR 76%, ∆12%; MR^4.5^ 42%, ∆9%). The estimated five-year OS rates in BFORE were high and similar in both treatment arms (bosutinib, 94.5% vs. imatinib, 94.6%), and comparable to those observed with nilotinib (300 mg nilotinib, 93.7% vs. imatinib, 91.7%) and dasatinib (dasatinib, 91% vs. imatinib, 90%).

Treatment-free remission is an emerging treatment goal of increasing importance, with several studies demonstrating that a substantial proportion of patients who achieve stable DMR maintain response after TKI discontinuation [12]. Few clinical trials have prospectively evaluated the incidence of patients achieving a sustained DMR. One study in de novo imatinib-treated patients reported a cumulative incidence of sustained (≥2 years) MR^4.5^ of 36.5% after eight years of treatment [13]. In a retrospective analysis of patients treated with frontline TKIs, with a median follow-up of 103 months, 47% of patients achieved a sustained (≥2 years) MR^4.5^ at any time [14]. In our study with a median follow-up of 55.2 months, a two-year sustained MR^4^ was achieved by 32.5% of patients treated with bosutinib versus 26.5% with imatinib. Some patients with a confirmed loss of MR subsequently regained the respective response with continued treatment, reflecting the fluctuations in *BCR::ABL1* often observed in patients before the achievement of a sustained DMR. This suggests that a follow-up ≤60 months may be insufficient to adequately assess the proportion of patients achieving sustained DMR. However, the rate observed with bosutinib by five years was similar to imatinib by eight years, suggesting (with acknowledgement of caution in comparison across studies) that treatment with second-generation TKIs may allow patients to achieve a sustained DMR faster, as would be expected based on the earlier achievement of DMR with second-generation TKIs [15]. This study also confirmed the achievement of *BCR::ABL1* transcript level ≤10% at three months as predictive of sustained MR^4^, as previously suggested in studies with other TKIs [15].

Despite the increasing interest in treatment-free remission, the majority of patients with CML will still require lifelong TKI treatment, and therefore preserving or improving health-related quality of life (HRQoL) remains an important consideration for treatment selection [16]. A previous analysis of BFORE demonstrated that HRQoL was maintained or improved compared with baseline after 12 months of bosutinib or imatinib treatment [17]. In addition, a pooled analysis of the bosutinib and imatinib arms showed that a better molecular response with tyrosine kinase inhibitor treatment was generally associated with improved HRQoL [8].

Safety data were consistent with the known safety profiles of bosutinib and imatinib in newly diagnosed patients with CP CML, and with second-line or later bosutinib treatment, with no new safety signals identified [4, 5, 7, 18–22]. The onset of TEAEs occurred primarily during the first year of treatment and they were generally manageable, with few new TEAEs (eg, effusion events) occurring in later years. In general, permanent treatment discontinuations due to AEs occurred early during treatment, most during the first year, confirming the importance of closely monitoring patients following initiation of treatment, particularly since rechallenge after temporary discontinuation due to toxicity has often been shown to be successful if management recommendations are followed [23, 24]. In patients receiving bosutinib or imatinib, there was a slight increase in the overall incidence of AEs of special interest; however, few patients in either arm discontinued treatment due to these AEs.

Liver function abnormalities were the most common AEs leading to treatment discontinuation of bosutinib. Although diarrhea was frequently reported in bosutinib-treated patients, few permanently discontinued treatment due to diarrhea, and the event rate was similar between treatment arms. Guidelines for the management of AEs occurring with bosutinib treatment have been published [23, 25].

As opposed to cerebrovascular and peripheral vascular events, which did not differ between treatments, cardiovascular TEAEs, although they remained low (≤5%) in both arms, were higher in the bosutinib versus imatinib arm (Table 3).

Exposure-adjusted incidence rates of cardiac and vascular TEAEs were slightly higher with bosutinib 400 mg once daily versus those observed in the phase 3 BELA trial of bosutinib 500 mg/d for newly diagnosed CP CML; however, patients in BFORE had a higher cardiovascular comorbidity burden at baseline compared with patients in BELA (Supplementary Tables 2, 9) [18, 19, 26].

Hyperlipidemia and hyperglycemia are major cardiovascular risk factors [27]. In this study, the overall rate of metabolic TEAEs was similar in the bosutinib and imatinib arms, with hyperlipidemia and hyperglycemia reported in ≤5% of patients in the bosutinib arm. These rates appear to be lower with bosutinib than those previously reported with nilotinib [10, 28].

Pleural effusions are more commonly associated with dasatinib; after five years of follow-up, 28% of patients receiving first-line dasatinib reported pleural effusions. Although their occurrence was higher with bosutinib than with imatinib, the incidence (6%) after five years appears to be lower compared with dasatinib. Importantly, pleural effusions can also first occur years after treatment (Table 3); however, they were generally manageable and rarely led to treatment discontinuation.

Renal dysfunction has been reported with imatinib and bosutinib and, to a lesser degree, with dasatinib [29, 30]. In this study, there was a similar decline in eGFR over time with both treatments; however, few patients in either treatment arm had a decline to Kidney Disease Improving Global Outcomes grade ≥3b, and ~50% of those patients had returned to grade ≤3a at their last assessment, suggesting a reversible mechanism.

Although the efficacy of bosutinib, nilotinib, and dasatinib is similar, bosutinib has a distinct safety profile, with a low incidence of some TEAEs compared with other TKIs (eg, vascular and effusion TEAEs) but higher incidence of other AEs (eg, diarrhea, liver) [6, 11]. A number of factors, including patients’ comorbidities and risk factors as well as the safety profile and schedule of administration of TKIs, should be considered when selecting the most appropriate TKI for the treatment of newly diagnosed patients with CP CML [23].

In conclusion, first-line bosutinib continued to show superior efficacy versus imatinib, with patients who received bosutinib achieving earlier and deeper MR. AEs were generally manageable, reversible, and consistent with the known safety profiles of both drugs. These results confirm the use of bosutinib as a standard of care in patients with newly diagnosed CP CML.

Information about this study in a plain language format is available in the supplementary materials.

## Supplementary information

Supplementary Materials

## Data Availability

Upon request, and subject to review, Pfizer will provide the data that support the findings of this study. Subject to certain criteria, conditions and exceptions, Pfizer may also provide access to the related individual de-identified participant data. See https://www.pfizer.com/science/clinical-trials/trial-data-and-results for more information.
