# Supplementary material for: Bosutinib versus imatinib for newly diagnosed chronic phase chronic myeloid leukemia: final results from the BFORE trial
Source: Leukemia. 2022 May 28;36(7):1825–33. doi: 10.1038/s41375-022-01589-y (PMC9252917; doi:10.1038/s41375-022-01589-y)
Supplement: Supplementary file 1 — Supplementary Materials [file 41375_2022_1589_MOESM1_ESM.pdf]

SUPPLEMENTARY MATERIALS

Study Investigators

United States	
Jitendra Gandhi	James McCloskey
Ilan Shapira	Jamile M. Shammo
Srinivas Devarakonda	Ehab L. Atallah
Peter D. Byeff	Michael W. Deininger[a]
Jorge Cortes[a,b]	Jennifer F. Carney
Stephen DiSean Kendall	Stefano Tarantolo
Jan Cerny	Bernard J. Poiesz
Valeriy Sedov	David Aboulafia
Luke Akard	Vivian Oehler
Ralph V. Boccia	Ajit S. Maniam
Jeffrey D. Neidhart	Stephen Medlin
Daniel Anderson	Christopher M. Reynolds
Andrew Kellum	Irum Khan
Rosalind Catchatourian	Vamsi K. Kota
Anthony Jaslowski	R. Timothy Webb
Moshe Talpaz	Thomas W. Butler
Satish Shah	Kerry Pulver
Saeeda Chowdhury	Salman Malad
Jason H. Mendler	Charles Kuzma
Michael E. Garrison	Brenda W. Cooper
David A. Rizzieri	Carrie L. Dul

Michael J. Mauro[a]	Aileen Go
Reinhold Munker	Kelly B. Pendergrass
Stefan Faderl	Alice Mims
Canada	
Ariah Schattner	Nizar Abdel-Samad
Rouslan Kotchetkov	Hadi Goubran
Jeffrey Lipton	Lambert Busque
Robert Delage	Brian Leber
Julie Stakiw	
South Africa	
Graham L. Cohen	Bernardo L. Rapoport
Lydia M. Dreosti	Nicolas Novitzky
Paul Ruff	
the Netherlands	
Jeroen J.W.M. Janssen	Edo Vellenga
Belgium	
Fritz Offner	Marie Lejeune
Gregor Verhoef	Gaetan Vanstraelen
Lucien Noens	
France	
Eric Jourdan	Philippe Rousselot
Mauricette Michallet	Viviane Dubruille
Francoise Huguet	Laurence Legros
José-Miguel Torregrosa-Diaz	Gabriel Etienne

Cristina Precupanu	Frederic Maloisel
Spain	
Valentin Garcia-Gutierrez	Luis Felipe Casado Montero
Juan Luis Steegmann	Guillermo Orti
Juan Carlos Hernandez-Boluda	Francisco Cervantes
Manuel Mateo Perez Encinas	Blanca Xicoy
Fermin Sánchez-Guijo	Ascension Pena
Santiago Osorio Prendes	Cristina Motllo
Pere Barba	
Finland	
Kimmo Porkka	Kari Remes
Hungary	
Zita Borbényi	Gyorgy Ujj
Árpád Illés	Miklós Egyed
Judit Demeter	Zoltán Gasztonyi
Ukraine	
Zvenyslava Maslyak	Svitlana Kalinina
Iryna Dyagil	Halyna Pylypenko
Nataliia Glushko	Grigoriy Rekhtman
Italy	
Fabio Ciceri	Fabrizio Pane
Carlo Gambacorti-Passerini[a]	Emilio Usala
Paolo Di Bartolomeo	Bruno Martino
Elisabetta Abruzzese	Gianantonio Rosti

Alberto Bosi	Alessandro Rambaldi
Fabio Stagno	Renato Bassan
Germana Beltrami	Emanuele Angelucci
Angelo Michele Carella	

Czech Republic	
Marek Trneny	Petra Belohlavkova
Jiri Mayer	Edgar Faber
Hana Klamova	

Slovakia	
Martin Mistrik	Ludmila Demitrovicova

United Kingdom	
Dragana Milojkovic	Jennifer Byrne
Jonathan Kell	Joanne Ewing
Graeme Smith	Ann Hunter
Richard Clark	Adam Mead
Sebastian Francis	Stewart McConnell
Sameer Tulpule	

Denmark	
Lene Udby	Marianne Severinsen
Jesper Stentoft	Hanne Vestergaard

Sweden	
Johan Richter	Kourosh Lotfi
Ulla Olsson Stroemberg	Berit Markevärn
Leif Stenke	Mats Bjoereman

Norway	
Henrik Hjorth-Hansen	Bjørn Tore Gjertsen
Poland	
Marek Hus	Slawomira Kyrcz-Krzemien
Tadeusz Robak	Tomasz Sacha
Andrzej Hellmann	Krzysztof Warzocha
Ewa Medras	Sebastian Grosicki
Kazimierz Kuliczkowski	
Germany	
Tim Brümmendorf[a,b]	Rainer Ordemann
Philipp le Coutre	Dietger Niederwieser
Gunhild von Arnsberg	Cornelius Waller
Andreas Hochhaus[a]	Dominik Wolf
Susanne Saußele	Jürgen Novotny
Kathleen Jentsch-Ullrich	
Mexico	
Rafael Hurtado Monroy	Enrique Baez de la Fuente
Australia	
John Catalano	David Ross
Shir-Jing Ho	Anthony Schwarer
Tim Hughes	
Singapore	
Charles Chuah[a]	Kiat Hoe Ong
Liang Piu Koh	

Thailand	
Tontanai Numbenjapon	Udomsak Bunworasate
Pimjai Niparuck	Surapol Issaragrisil
Adisak Tantiworawit	
Korea	
Dong-Wook Kim[a]	Dae Young Zang
Young Rok Do	Jae-Yong Kwak
Suk Joong Oh	Sung-Hyun Kim
Taiwan	
Ken-Hong Lim	Lee-Yung Shih
Su-Peng Yeh	Ta-Chih Liu
Cheng-Hwai Tzeng	Yin-Hsun Feng
Jin-Hwang Liu	
Israel	
Noam Benyamini	Pia Raanani

[a]Study Management Committee member.

[b]Coordinating investigator.

SUPPLEMENTARY METHODS

Efficacy and safety assessments

Efficacy was evaluated by standard criteria, including molecular response (MR) reported on the international scale, cytogenetic response, and hematologic response.[1] Mutation analysis was performed in case of suboptimal response or treatment failure and at end of treatment. MRs assessed by real-time quantitative polymerase chain reaction and mutation analysis using Sanger sequencing were performed at a central laboratory (MolecularMD, Portland, OR, USA).

Safety was assessed as previously reported.[1] Frequency and characteristics of adverse events (AEs) of special interest were analyzed by selecting Medical Dictionary for Regulatory Activities (MedDRA) system organ class high-level group terms (HLGT), high-level terms (HLT), and preferred terms (PT), and standardized MedDRA queries (SMQ) to generate treatment-emergent AE (TEAE) clusters. MedDRA terms included in the TEAE clusters were:

- **Cardiac.** *HLGT:* Cardiac arrhythmias, Heart failures; *PT:* Cardiac death, Sudden cardiac death, Sudden death, Ejection fraction decreased; *SMQ:* Torsade de pointes / QT prolongation (narrow).
- **Edema.** *PT:* Contains Edema or Weight increased.
- **Effusion.** *PT:* Pericardial effusion or Pleural effusion.
- **Gastrointestinal.** *PT:* Nausea, Regurgitation, Retching, Vomiting, Vomiting projectile, Diarrhea, Defecation urgent, Frequent bowel movements, Gastrointestinal hypermotility.
- **Hypertension.** *HLGT:* Vascular hypertension disorders; *PT:* Blood pressure (BP) abnormal, BP ambulatory abnormal, BP ambulatory increased, BP diastolic abnormal, BP diastolic increased, BP increased, BP systolic abnormal, BP systolic increased.

- **Liver.** *Sub-SMQ (Narrow):* Cholestasis and jaundice of hepatic origin; Hepatic failure, fibrosis, and cirrhosis and other liver damage–related conditions; Hepatitis, non-infectious; *PT:* Alanine aminotransferase (ALT) abnormal, ALT increased, Aspartate aminotransferase (AST) abnormal, AST increased, Bilirubin conjugated abnormal, Bilirubin conjugated increased, Blood bilirubin abnormal, Blood bilirubin increased, Blood bilirubin unconjugated increased, Hepatic enzyme abnormal, Hepatic enzyme increased, Hepatic function abnormal, Hyperbilirubinemia, Hypertransaminasemia, Liver function test abnormal, Liver function test increased, Transaminase abnormal, Transaminase increased, Blood alkaline phosphatase abnormal, Blood alkaline phosphatase increased.
- **Metabolic.** Diabetes mellitus (HLT: Diabetes mellitus [including subtypes]), Hypercholesterolemia (*PT*: Hypercholesterolemia, blood cholesterol increased), Hyperlipidemia (*PT*: Hyperlipidemia, lipids increased), Hypertriglyceridemia (*PT*: Hypertriglyceridemia, blood triglycerides increased), Hyperglycemia (*PT*: Hyperglycemia, blood glucose increased).
- **Musculoskeletal.** *PT:* Arthralgia, Muscle spasms, Pain in extremity, Myalgia, Back pain, Bone pain, Musculoskeletal pain, Musculoskeletal stiffness, Joint stiffness, Musculoskeletal discomfort, Muscle fatigue, Muscle tightness, Muscle twitching, Joint swelling, Muscular weakness, Arthritis, Muscle discomfort, Muscular weakness.
- **Myelosuppression.** *SMQ (Narrow):* Hematopoietic cytopenias affecting more than one type of blood cell, Hematopoietic erythropenia, Hematopoietic leukopenia, Hematopoietic thrombocytopenia; *PT:* Bone marrow toxicity, Hematocrit decreased, Hemoglobin decreased, Hematotoxicity, Anemia.
- **Rash.** *HLT*: Rashes, Eruptions and exanthemas not elsewhere classified (NEC), Erythemas, Acnes, Dermatitis and eczema.

- **Renal**. *HLT:* Renal failure and impairment; *PT:* Blood creatinine abnormal, Blood creatinine increased, Creatinine renal clearance abnormal, Creatinine renal clearance decreased, Glomerular filtration rate abnormal, Glomerular filtration rate decreased.
- **Vascular** includes MedDRA terms for cardiovascular, cerebrovascular, and peripheral vascular TEAEs:
  - ***Cardiovascular*****:** *HLGT:* Coronary artery disorders; *HLT:* Arterial therapeutic procedures (excluding aortic), Vascular imaging procedures NEC, Vascular therapeutic procedures NEC; *PT:* Transcatheter arterial chemoembolization.
  - ***Cerebrovascular*****:** *HLT:* Central nervous system (CNS) hemorrhages and cerebrovascular accidents, CNS vascular disorders NEC, Transient cerebrovascular events.
  - ***Peripheral vascular****: HLGT:* Arteriosclerosis, stenosis, vascular insufficiency, and necrosis, Embolism and thrombosis; *HLT:* Non-site-specific vascular disorders NEC, Peripheral vascular disorders NEC (excluding PTs flushing and hot flush); *PT:* Intestinal ischemia.

In all analyses the following MedDRA PTs were clustered for cytopenias:

- Anemia: Anemia, Hemoglobin decreased.
- Neutropenia: Neutropenia, Neutrophil count decreased.
- Leukopenia: Leukopenia, White blood cell count decreased.
- Thrombocytopenia: Thrombocytopenia, Platelet count decreased.
- Lymphopenia: Lymphopenia, Lymphocyte count decreased.

Electrocardiograms (ECGs) were performed, in triplicate, predose on day 1, and on days 28, 56, and 84, and end of treatment. ECGs were also performed as clinically indicated.

Estimated glomerular filtration rate (eGFR) was calculated using the Modification of Diet in Renal Disease formula and graded on the basis of the Kidney Disease Improving Global

Outcomes criteria.[2]

Statistical analyses

Exposure-adjusted incidence of TEAEs was calculated as the number of patients with TEAEs per total patient-year (sum of total time to first TEAE for all patients with TEAEs plus treatment duration for patients without TEAEs). Exact Poisson 95% confidence intervals (CIs) were provided.

Distribution-free CIs for the median GFR change from baseline based on Hahn and Meeker order statistics (ranks) were provided.

Time-to-event endpoints

Time-to-event endpoints, except for duration of response (DOR) and overall survival (OS), were estimated using cumulative incidence, adjusting for the competing risk of treatment discontinuation without the event (event-free survival [EFS] analyzed as time to progression or death); DOR and OS were estimated using the Kaplan–Meier method. Patients without events were censored at the last valid assessment for the respective time-to-event endpoint. Patients without OS events were censored at last known alive date. Standard errors for 95% CI rates were constructed using a log(–log) transformation. Odds ratio and Mantel–Haenszel 95% CI for bosutinib versus imatinib were adjusted for Sokal risk group and geographic region unless specified otherwise. Hazard ratio and corresponding 95% CI for bosutinib versus imatinib for DOR and OS were based on a Cox proportional hazards model and, for EFS and time to response, on a proportional subdistribution hazards model (both stratified by Sokal risk group and geographic region). *P* values were based on a stratified Cochran–Mantel–Haenszel test for response, a stratified log-rank test for OS, and a stratified Gray's test for EFS.

Multivariable proportional subdistribution hazards models predicting time to sustained MR[4] (intent-to-treat [ITT] population) and time to first cardiac, vascular, and effusion TEAE and grade ≥3b eGFR (safety population) were performed. Patients without events were censored at last valid molecular assessment for sustained MR[4] and last dose date for safety outcomes.

Control of type 1 error rate

To address the issue of multiplicity, secondary endpoints were grouped into short-term and long-term families with the long-term family evaluated at the end of the study. Given the primary endpoint analysis was significant, each endpoint of the short-term family was tested via the Bonferroni's procedure at the 1-sided 0.0125 significance level. Complete cytogenetic response (CCyR) by 12 months was significant at the time of the primary endpoint analysis. If either major molecular response (MMR) by 18 months or CCyR by 12 months in the short-term family was significant, the long-term family endpoints were tested via the Holm stepwise test. The long-term secondary family endpoints (EFS, OS) were tested at either the 1-sided 0.025 alpha (if both CCyR by 12 months and MMR by 18 months were significant at 0.0125) or the 1-sided 0.0125 (if only one of the two endpoints was significant at 0.0125). This approach to multiple testing strongly controlled the overall type 1 error rate at the 1-sided 0.025 significance level. All family-wise comparisons were based on the modified ITT population (where applicable), which was consistent with the ITT population. No additional adjustment for multiple comparisons was made.

REFERENCES

1. Cortes JE, Gambacorti-Passerini C, Deininger MW, Mauro MJ, Chuah C, Kim DW, et al. Bosutinib versus imatinib for newly diagnosed chronic myeloid leukemia: results from the randomized BFORE trial. J Clin Oncol. 2018;36:231-7.

2. Kidney Disease: Improving Global Outcomes (KDIGO): KDIGO 2012 clinical practice guideline for the evaluation and management of chronic kidney disease. Kidney Int Suppl 2013;3(1):1-150. doi 10.1038/kisup.2012.72. https://kdigo.org/wp-content/uploads/2017/02/KDIGO_2012_CKD_GL.pdf. Accessed 19 Mar, 2021.

Supplementary Table 1 Demographic and baseline characteristics in patients randomized to bosutinib and imatinib.

**Characteristic**	**Bosutinib** *n* = **268**	**Imatinib** *n* = **268**
Age, median (range), y	53 (18–84)	53 (19–84)
Sex, *n* (%)		
Male	156 (58.2)	155 (57.8)
Female	112 (41.8)	113 (42.2)
Race, *n* (%)		
White	211 (78.7)	206 (76.9)
Asian	33 (12.3)	34 (12.7)
Black	10 (3.7)	10 (3.7)
Other[a]	14 (5.2)	18 (6.7)
Sokal risk group at screening, *n* (%)		
Low	95 (35.4)	106 (39.6)
Intermediate	117 (43.7)	105 (39.2)
High	56 (20.9)	57 (21.3)
ECOG performance status, *n* (%)		

0	195 (72.8)	194 (72.4)
1	73 (27.2)	74 (27.6)
Philadelphia chromosome status, *n* (%)[b]		
Positive	249 (92.9)	244 (91.0)
Negative	6 (2.2)	6 (2.2)
Unknown	13 (4.9)	18 (6.7)
BCR::ABL1 transcript type, *n* (%)		
Typical (e13a2/e14a2)	265 (98.9)	263 (98.1)
Atypical[c]	3 (1.1)	5 (1.9)

ECOG Eastern Cooperative Oncology Group, *Ph+* Philadelphia chromosome–positive.

[a]One patient with missing race in the imatinib arm is included in the "other" category.

[b]Negative was defined as 0 Ph+ chromosomes of ≥10 metaphases at baseline; unknown includes missing data or 0 Ph+ chromosomes of <10 metaphases at baseline.

[c]Includes one patient in the bosutinib arm with typical *BCR::ABL1* transcripts detected postbaseline and one patient in the imatinib arm with no *BCR::ABL1* transcripts detected at baseline who did not receive study drug.

Supplementary Table 2 Baseline cardiovascular risk factors in patients receiving bosutinib and imatinib.

	**BFORE trial**		**BELA for reference**[a]	
**Cardiovascular risk factors, *n* (%)**	**Bosutinib 400 mg QD *n* = 268**	**Imatinib 400 mg QD *n* = 265**	**Bosutinib 500 mg QD *n* = 248**	**Imatinib 400 mg QD *n* = 251**
History of cardiac disorders[b]	19 (7.1)	17 (6.4)	5 (2.0)	13 (5.2)
History of vascular disorders[c]	35 (13.1)	42 (15.8)	17 (6.9)	18 (7.2)
History of hypertension[d]	91 (34.0)	81 (30.6)	48 (19.4)	58 (23.1)
History of tobacco use[e]	7 (2.6)	2 (0.8)	5 (2.0)	0
History of hyperlipidemia[f]	45 (16.8)	50 (18.9)	26 (10.5)	32 (12.7)
History of diabetes mellitus[g]	31 (11.6)	33 (12.5)	14 (5.6)	17 (6.8)
BMI >30 kg/m^2	62 (23.1)	58 (21.9)	38 (15.3)	39 (15.5)
Age ≥65 years	53 (19.8)	46 (17.4)	28 (11.3)	26 (10.4)
History of only 1 of above	61 (22.8)	63 (23.8)	44 (17.7)	47 (18.7)
History of only 2 of above	38 (14.2)	39 (14.7)	32 (12.9)	28 (11.2)
History of ≥3 of above	56 (20.9)	47 (17.7)	20 (8.1)	28 (11.2)

History of ≥1 risk factor	155 (57.8)	149 (56.2)	96 (38.7)	103 (41.0)

BMI body mass index, *BP* blood pressure, *CNS* central nervous system, *HLGT* high-level group term, *HLT* high-level term, *MedDRA* Medical Dictionary for Regulatory Activities, *NEC* not elsewhere classified, *PT* preferred term, *QD* once daily, *SMQ* standardized MedDRA query.

[a]Based on the final database snapshot in 2015.

[b]Includes the MedDRA *HLGT*: Cardiac arrhythmias, Heart failures; *PT*: Cardiac death, Sudden cardiac death, Sudden death, Ejection fraction decreased; *SMQ*: Torsade de pointes / QT prolongation (narrow).

[c]Includes the MedDRA *HLGT*: Coronary artery disorders; Arteriosclerosis, stenosis, vascular insufficiency, and necrosis; Embolism and thrombosis; *HLT*: Arterial therapeutic procedures (excluding aortic), CNS hemorrhages and cerebrovascular accidents, CNS vascular disorders NEC, Non-site-specific vascular disorders NEC, Peripheral vascular disorders NEC (excluding PTs flushing and hot flush), Transient cerebrovascular events, Vascular imaging procedures NEC, Vascular therapeutic procedures NEC; *PT*: Intestinal ischemia, Transcatheter arterial chemoembolization.

[d]Includes the MedDRA *HLGT*: Vascular hypertension disorders; *PT*: BP abnormal, BP ambulatory abnormal, BP ambulatory increased, BP diastolic abnormal, BP diastolic increased, BP increased, BP systolic abnormal, BP systolic increased.

[e]Includes the MedDRA *PT*: Tobacco user, Ex-tobacco user, and Tobacco abuse.

[f]Includes the MedDRA *HLGT:* Lipid metabolism disorders; *PT:* Blood cholesterol increased, Blood triglycerides increased, Lipids increased.

[g]Includes the MedDRA *HLT:* Diabetes mellitus (including subtypes), Hyperglycemic conditions NEC; *PT:* Blood glucose increased, Glycosylated hemoglobin increased.

Supplementary Table 3 Multivariable subdistributional hazards model for time to sustained MR^4.

Parameter	Test vs. reference	1-year sustained MR^4 HR (95% CI)	2-year sustained MR^4 HR (95% CI)
Age, y	1 unit increase	1.00 (0.99−1.01)	1.00 (0.99–1.01)
Race	White vs. other	1.16 (0.71− 1.90)	1.11 (0.64–1.92)
Gender	Male vs. female	1.06 (0.80−1.40)	1.16 (0.83–1.62)
ECOG PS	>0 vs. 0	0.79 (0.57−1.11)	0.63 (0.42–0.96)
Geographical region[a]	Region 2 vs. 1	1.23 (0.90−1.68)	1.38 (0.97–1.98)
	Region 3 vs. 1	1.19 (0.66−2.13)	1.27 (0.66–2.43)
Sokal risk group	Low vs. high	0.96 (0.64−1.44)	0.95 (0.60–1.51)
	Intermediate vs. high	0.95 (0.64−1.41)	0.93 (0.60–1.45)
Treatment group	Bosutinib vs. imatinib	1.09 (0.83−1.45)	1.10 (0.79–1.54)
BCR::ABL1 transcript levels ≤10% at 3 months[b]	≤10% vs. no	3.58 (2.46−5.22)	3.88 (2.46–6.14)
First dose reduction to 300 mg[b]	Dose reduction vs. no reduction	0.79 (0.57−1.11)	0.84 (0.57–1.24)
CCI score[c]	CCI >2 vs. 2	1.12 (0.80−1.57)	1.18 (0.79–1.76)

HR <1 favors the reference group.

CCI Charlson Comorbidity Index score, *CI* confidence interval, *ECOG PS* Eastern Cooperative Oncology Group performance status, *HR* hazard ratio, *MR* molecular response.

[a]Region 1: United States, Canada, Western Europe; Region 2: Eastern Europe, Latin America, South America; Region 3: Rest of World.

[b]Time-dependent covariate.

[c]CCI score was calculated without the age component.

Supplementary Table 4 Reason of death in patients receiving bosutinib or imatinib.

Cause of death	Bosutinib<br>*n* = 268	Imatinib<br>*n* = 265
Any reason (any time on study)	14 (5.2)	14 (5.3)
AE unrelated to study drug	7 (2.6)	3 (1.1)
AE related to study drug	0	1 (0.4)
Disease progression	3 (1.1)	4 (1.5)
Other	3 (1.1)	6 (2.3)
Unknown	1 (0.4)	0
Any reason (within 28 days of last dose)	3 (1.1)	4 (1.5)
AE unrelated to study drug	3 (1.1)	2 (0.8)
AE related to study drug	0	1 (0.4)
Disease progression	0	1 (0.4)

AE adverse event.

Supplementary Table 5 Treatment-emergent adverse events.[a]

n (%)	**Bosutinib (*n* = 268)**		**Imatinib (*n* = 265)**	
	**Any grade**	**Grade 3/4**	**Any grade**	**Grade 3/4**
Any TEAE	265 (98.9)	197 (73.5)	262 (98.9)	151 (57.0)
Diarrhea	201 (75.0)	24 (9.0)	107 (40.4)	3 (1.1)
Nausea	100 (37.3)	0	112 (42.3)	0
Thrombocytopenia	96 (35.8)	38 (14.2)	53 (20.0)	16 (6.0)
ALT increased	90 (33.6)	56 (20.9)	16 (6.0)	4 (1.5)
AST increased	69 (25.7)	28 (10.4)	18 (6.8)	5 (1.9)
Rash	62 (23.1)	2 (0.7)	39 (14.7)	3 (1.1)
Abdominal pain	61 (22.8)	5 (1.9)	25 (9.4)	1 (0.4)
Anemia	59 (22.0)	12 (4.5)	60 (22.6)	15 (5.7)
Headache	59 (22.0)	3 (1.1)	41 (15.5)	3 (1.1)
Fatigue	57 (21.3)	2 (0.7)	54 (20.4)	0
Lipase increased	56 (20.9)	36 (13.4)	30 (11.3)	15 (5.7)
Vomiting	55 (20.5)	3 (1.1)	54 (20.4)	0
Arthralgia	48 (17.9)	3 (1.1)	49 (18.5)	1 (0.4)
Pyrexia	46 (17.2)	3 (1.1)	30 (11.3)	0
Upper respiratory tract infection	37 (13.8)	1 (0.4)	33 (12.5)	0
Nasopharyngitis	36 (13.4)	1 (0.4)	30 (11.3)	0
Constipation	36 (13.4)	0	17 (6.4)	0
Asthenia	34 (12.7)	0	24 (9.1)	1 (0.4)
Neutropenia	33 (12.3)	20 (7.5)	61 (23.0)	36 (13.6)
Back pain	32 (11.9)	1 (0.4)	25 (9.4)	1 (0.4)
Cough	30 (11.2)	0	26 (9.8)	0
Decreased appetite	30 (11.2)	1 (0.4)	17 (6.4)	0

Pruritus	30 (11.2)	1 (0.4)	10 (3.8)	0
Dyspnea	29 (10.8)	2 (0.7)	15 (5.7)	2 (0.8)
Abdominal pain upper	28 (10.4)	0	27 (10.2)	1 (0.4)
Urinary tract infection	27 (10.1)	1 (0.4)	20 (7.5)	2 (0.8)
Hypertension	26 (9.7)	12 (4.5)	29 (10.9)	12 (4.5)
Pain in extremity	26 (9.7)	2 (0.7)	39 (14.7)	0
Edema peripheral	20 (7.5)	0	43 (16.2)	1 (0.4)
Leukopenia	18 (6.7)	4 (1.5)	34 (12.8)	12 (4.5)
Blood creatine phosphokinase increased	14 (5.2)	5 (1.9)	33 (12.5)	10 (3.8)
Myalgia	13 (4.9)	1 (0.4)	48 (18.1)	2 (0.8)
Muscle spasms	10 (3.7)	0	81 (30.6)	1 (0.4)
Periorbital edema	4 (1.5)	0	44 (16.6)	0

ALT alanine aminotransferase, *AST* aspartate aminotransferase, *TEAE* treatment-emergent adverse event.

[a]Any grade TEAEs occurring in ≥10% and/or grade 3 or 4 TEAEs occurring in ≥5% of patients in the bosutinib or imatinib arm.

Supplementary Table 6 Laboratory abnormalities occurring in ≥30% of patients in the bosutinib or imatinib arm.

	Bosutinib (*n* = 268)		Imatinib (*n* = 265)	
**Laboratory abnormality, *n* (%)**	**All grades**	**Grade 3/4**	**All grades**	**Grade 3/4**
Increased creatinine	252 (94.0)	3 (1.1)	260 (98.1)	2 (0.8)
Decreased hemoglobin	238 (88.8)	23 (8.6)	239 (90.2)	18 (6.8)
Decreased lymphocytes	225 (84.0)	33 (12.3)	216 (81.5)	37 (14.0)
Increased ALT	183 (68.3)	70 (26.1)	75 (28.3)	8 (3.0)
Decreased platelets	183 (68.3)	38 (14.2)	158 (59.6)	16 (6.0)
Increased glucose	153 (57.1)	8 (3.0)	172 (64.9)	9 (3.4)
Increased AST	150 (56.0)	36 (13.4)	78 (29.4)	9 (3.4)
Decreased calcium	147 (54.9)	4 (1.5)	152 (57.4)	3 (1.1)
Decreased phosphate	144 (53.7)	25 (9.3)	184 (69.4)	55 (20.8)
Increased lipase	141 (52.6)	51 (19.0)	92 (34.7)	21 (7.9)
Decreased leukocytes	134 (50.0)	17 (6.3)	186 (70.2)	22 (8.3)
Decreased neutrophils	113 (42.2)	25 (9.3)	171 (64.5)	52 (19.6)
Increased alkaline phosphatase	110 (41.0)	0	114 (43.0)	1 (0.4)
Increased creatine kinase	97 (36.2)	8 (3.0)	172 (64.9)	14 (5.3)
Increased amylase	85 (31.7)	9 (3.4)	47 (17.7)	6 (2.3)
Decreased potassium	34 (12.7)	6 (2.2)	80 (30.2)	8 (3.0)

ALT alanine aminotransferase, *AST* aspartate aminotransferase.

Supplementary Table 7 Any grade AEs leading to permanent treatment discontinuation in ≥1% of patients receiving bosutinib or imatinib.

***n* (%)**	**Bosutinib** ***n* = 268**	**Imatinib** ***n* = 265**
Any AE	68 (25.4)	38 (14.3)
ALT increased	13 (4.9)	0
AST increased	7 (2.6)	0
Lipase increased	5 (1.9)	2 (0.8)
Diarrhea	4 (1.5)	3 (1.1)
Thrombocytopenia	3 (1.1)	4 (1.5)
Neutropenia	3 (1.1)	1 (0.4)
Muscle spasms	0	3 (1.1)
Myalgia	0	3 (1.1)

AE adverse event, *ALT* alanine aminotransferase, *AST* aspartate aminotransferase.

Supplementary Fig. 1 Any grade AEs leading to permanent treatment discontinuation, by year.

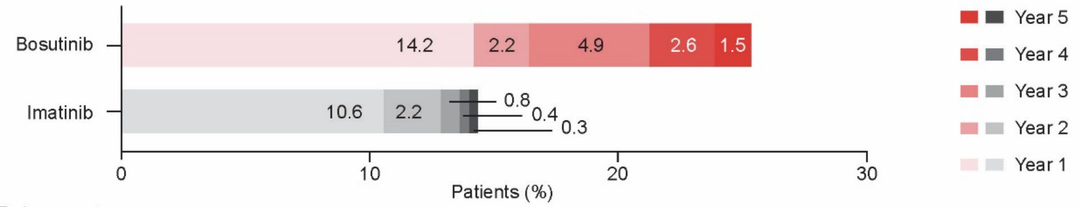

AE adverse event.

Supplementary Table 8 Multivariable subdistributional hazards model for time to initial cardiac, vascular, and effusion TEAEs.

**Parameter**[a]	**HR (95% CI)**		
	**Cardiac**	**Vascular**	**Effusion**
Age, y	1.04 (1.02–1.07)	1.01 (0.98–1.04)	1.08 (1.01–1.16)
Sex (male, female)	0.87 (0.48–1.57)	1.49 (0.60–3.66)	1.36 (0.49–3.83)
Race (White, other)	1.38 (0.54–3.50)	1.74 (0.57–5.32)	0.55 (0.20–1.55)
ECOG PS (>0, 0)	1.57 (0.85–2.87)	0.40 (0.13–1.29)	1.16 (0.46–2.93)
BMI, kg/m^2	1.01 (0.96–1.08)	1.00 (0.94–1.07)	0.98 (0.90–1.07)
History of (yes, no)			
Tobacco use[b]	3.31 (0.80–13.74)	2.86 (0.54–15.02)	<0.001 (<0.001–<0.001)
Cardiovascular disease[c]	N/A	N/A	1.11 (0.36–3.48)
Vascular disease[d]	0.70 (0.32–1.54)	4.76 (1.85–12.28)	N/A
Hypertension[e]	1.05 (0.55–2.03)	0.56 (0.20–1.57)	3.57 (0.91–14.01)
Hyperlipidemia[f]	0.83 (0.42–1.63)	0.96 (0.30–3.14)	2.63 (0.97–7.14)
Diabetes mellitus[g]	0.93 (0.46–1.86)	3.05 (1.03–9.07)	N/A
Pulmonary disease[h]	N/A	N/A	3.74 (1.73–8.08)
Cardiac disease[i]	3.45 (1.60–7.41)	0.48 (0.08–2.86)	0.24 (0.02–2.46)
Hypertension TEAE[j]	3.08 (1.24–7.68)	2.13 (0.48–9.45)	N/A
Vascular TEAE[j]	5.10 (1.51–17.23)	N/A	N/A
Cardiac TEAE[j]	N/A	7.94 (2.37–26.58)	N/A
Treatment (bosutinib, imatinib)	0.91 (0.47–1.74)	2.23 (0.97–5.09)	2.98 (1.08–8.19)

BMI body mass index, *BP* blood pressure, *CI* confidence interval, *CNS* central nervous system, *ECOG PS* Eastern Cooperative Oncology Group performance status, *HLGT* high-level group term, *HLT* high-level term, *HR* hazard ratio, *MedDRA* Medical Dictionary for Regulatory Activities, *N/A* not applicable, *NEC* not elsewhere classified, *PT* preferred term, *SMQ* standardized MedDRA query, *TEAE* treatment-emergent adverse event.

[a]Continuous variables are based on a 1-unit increase. For categorical variables, the level used as reference for each covariate was the second category listed for the covariate. HR >1 favors the reference group.

[b]Includes the MedDRA *PT*: Tobacco user, Ex-tobacco user, and Tobacco abuse.

[c]Includes the MedDRA *HLGT:* Coronary artery disorders; *HLT*: Arterial therapeutic procedures (excluding aortic), Vascular imaging procedures NEC, Vascular therapeutic procedures NEC; *PT:* Transcatheter arterial chemoembolization.

[d]Includes the MedDRA *HLGT*: Coronary artery disorders; Arteriosclerosis, stenosis, vascular insufficiency, and necrosis; Embolism and thrombosis; *HLT*: Arterial therapeutic procedures (excluding aortic), CNS hemorrhages and cerebrovascular accidents, CNS vascular disorders NEC, Non-site-specific vascular disorders NEC, Peripheral vascular disorders NEC (excluding PTs flushing and hot flush), Transient cerebrovascular events, Vascular imaging procedures NEC, Vascular therapeutic procedures NEC; *PT*: Intestinal ischemia, Transcatheter arterial chemoembolization.

[e]Includes the MedDRA *HLGT*: Vascular hypertension disorders; *PT*: BP abnormal, BP ambulatory abnormal, BP ambulatory increased, BP diastolic abnormal, BP diastolic increased, BP increased, BP systolic abnormal, BP systolic increased.

[f]Includes the MedDRA *HLGT:* Lipid metabolism disorders; *PT:* Blood cholesterol increased, Blood triglycerides increased, Lipids increased.

[g]Includes the MedDRA *HLT:* Diabetes mellitus (including subtypes), Hyperglycemic conditions NEC; *PT:* Blood glucose increased, Glycosylated hemoglobin increased.

[h]Includes the MedDRA *HLGT*: Bronchial disorders (excluding neoplasms), Lower respiratory tract disorders (excluding obstruction and infection), Pleural disorders, Pulmonary vascular disorders, Respiratory disorders NEC; *PT*: Pleuropericarditis, Pulmonary arterial pressure increased.

[i]Includes the MedDRA *HLGT*: Cardiac arrhythmias, Heart failures; *PT*: Cardiac death, Sudden cardiac death, Sudden death, Ejection fraction decreased; *SMQ*: Torsade de pointes / QT prolongation (narrow).

[j]Time-dependent covariates.

Supplementary Table 9 Exposure-adjusted incidence of select any grade AEs of special interest.

Exposure-adjusted incident rate (95% CI)[b]	BFORE trial		BELA for reference[a]	
	Bosutinib 400 mg QD $n = 268$	Imatinib 400 mg QD $n = 265$	Bosutinib 500 mg QD $n = 248$	Imatinib 400 mg QD $n = 251$
Cardiac TEAEs[c]	0.031 (0.020–0.046)	0.029 (0.018–0.043)	0.024 (0.015–0.037)	0.019 (0.010–0.031)
Vascular TEAEs[d]	0.023 (0.014–0.036)	0.011 (0.005–0.021)	0.017 (0.009–0.028)	0.011 (0.005–0.020)
Cardiovascular	0.015 (0.008–0.026)	0.001 (<0.001–0.007)	0.008 (0.003–0.017)	0.006 (0.002–0.014)
Cerebrovascular	0.002 (<0.001–0.008)	0.004 (0.001–0.010)	0.002 (<0.001–0.008)	0.002 (<0.001–0.009)
Peripheral vascular	0.007 (0.002–0.015)	0.007 (0.003–0.016)	0.006 (0.002–0.014)	0.002 (<0.001–0.009)
Effusion TEAEs[e]	0.019 (0.011–0.030)	0.007 (0.003–0.016)	0.025 (0.016–0.038)	0.001 (<0.001–0.007)
Pleural effusion	0.016 (0.009–0.027)	0.006 (0.002–0.014)	0.023 (0.014–0.035)	0.001 (<0.001–0.007)
Pericardial effusion	0.006 (0.002–0.013)	0.001 (<0.001–0.007)	0.007 (0.003–0.015)	0.000 (0.000–0.004)

AE adverse event, *CI* confidence interval, *CNS* central nervous system, *HLGT* high-level group term, *HLT* high-level term, *MedDRA* Medical Dictionary for Regulatory Activities, *NEC* not elsewhere classified, *PT* preferred term, *QD* once daily, *SMQ* standardized MedDRA query, *TEAE* treatment-emergent adverse event.

[a]Based on the final database snapshot in 2015.

[b]Exposure-adjusted incidence rate calculated as the number of patients with TEAEs / total patient-year, where total patient-year = sum of total time to first TEAE for all patients with TEAEs and treatment duration for patients without TEAEs.

[c]Includes the MedDRA *HLGT*: Cardiac arrhythmias, Heart failures; *PT*: Cardiac death, Sudden cardiac death, Sudden death, Ejection fraction decreased; *SMQ*: Torsade de pointes / QT prolongation (narrow).

[d]Vascular includes MedDRA terms for cardiovascular, cerebrovascular, and peripheral vascular TEAEs:

- ***Cardiovascular*****:** *HLGT:* Coronary artery disorders; *HLT:* Arterial therapeutic procedures (excluding aortic), Vascular imaging procedures NEC, Vascular therapeutic procedures NEC; *PT:* Transcatheter arterial chemoembolization.
- ***Cerebrovascular*****:** *HLT*: CNS hemorrhages and cerebrovascular accidents, CNS vascular disorders NEC, Transient cerebrovascular events.
- ***Peripheral vascular:*** *HLGT*: Arteriosclerosis, stenosis, vascular insufficiency, and necrosis; Embolism and thrombosis; *HLT:* Non-site-specific vascular disorders NEC, Peripheral vascular disorders NEC (excluding PTs flushing and hot flush); *PT:* Intestinal ischemia.

[e]Includes the MedDRA *PT*: Pericardial effusion, Pleural effusion.

Supplementary Fig. 2 Median change from baseline. A eGFR. **B** Blood creatinine. **C** Blood urea nitrogen over time in patients receiving bosutinib or imatinib.

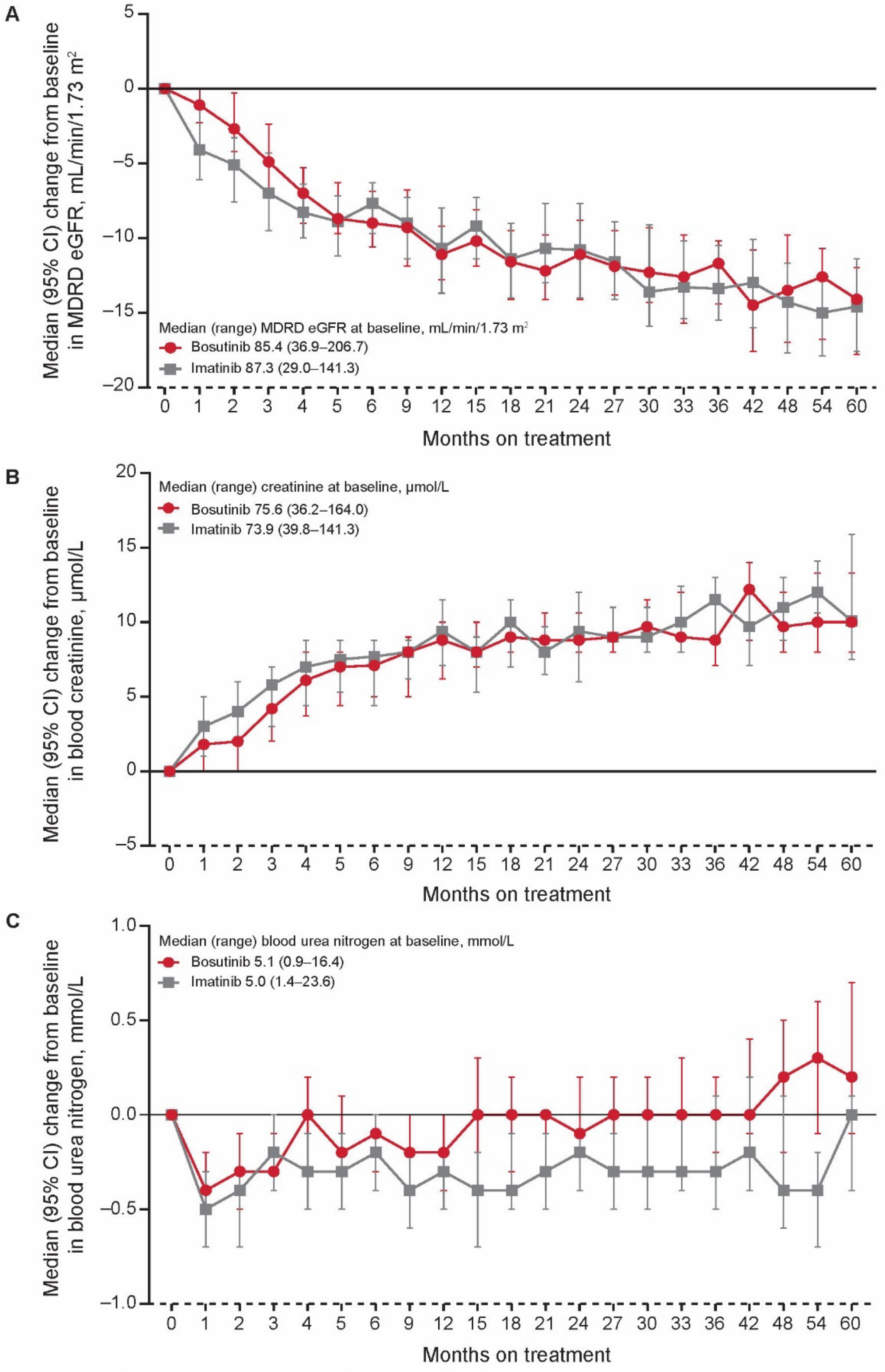

CI confidence interval, *eGFR* estimated glomerular filtration rate, *MDRD* Modification of Diet in Renal Disease.

Supplementary Table 10 Maximum blood urea nitrogen on or after initial eGFR <45 vs. on-treatment for eGFR ≥45 mL/min/1.73 m^2 in patients receiving bosutinib or imatinib.

	**Bosutinib**		**Imatinib**	
	**eGFR <45[a]** *n* **= 37**	**eGFR ≥45[a]** *n* **= 229**	**eGFR <45[a]** *n* **= 23**	**eGFR ≥45[a]** *n* **= 241**
Blood urea nitrogen (mmol/L) median (range)	11.3 (6.7–49.6)	6.4 (1.9–48.6)	7.9 (3.4–28.2)	6.1 (1.2–86.0)

eGFR estimated glomerular filtration rate.

[a]eGFR measured in mL/min/1.73 m^2.

Supplementary Table 11 Multivariable subdistributional hazards model for time to initial grade ≥3b eGFR.

**Parameter**[a]	**HR**	**95% CI**
Age, y	1.06	1.03–1.08
Sex (male, female)	0.86	0.50–1.48
Race (White, other)	0.49	0.26–0.94
ECOG PS (>0, 0)	2.50	1.49–4.20
BMI, kg/m^2	1.02	0.96–1.08
Baseline eGFR, mL/min/1.73 m^2	0.94	0.91–0.96
History of (yes, no)		
Tobacco use[b]	<0.001	<0.001–<0.001
Cardiovascular disease[c]	0.75	0.36–1.54
Hypertension[d]	1.10	0.60–2.04
Renal disease[e]	5.74	2.68–12.30
Diabetes mellitus[f]	2.78	1.58–4.89
Hyperuricemia[g]	0.39	0.09–1.75
Heart failure[h]	1.30	0.35–4.87
Treatment (bosutinib, imatinib)	1.39	0.85–2.30

BMI body mass index, *BP* blood pressure, *CI* confidence interval, *ECOG PS* Eastern Cooperative Oncology Group performance status, *eGFR* estimated glomerular filtration rate, *HLGT* high-level group term, *HLT* high-level term, *HR* hazard ratio, *MedDRA* Medical Dictionary for Regulatory Activities, *NEC* not elsewhere classified, *PT* preferred term.

[a]Continuous variables are based on a 1-unit increase. For categorical variables, the level used as reference for each covariate was the second category listed for the covariate. HR >1 favors the reference group.

[b]Includes the MedDRA *PT*: Tobacco user, Ex-tobacco user, and Tobacco abuse.

[c]Includes the MedDRA *HLGT:* Coronary artery disorders; *HLT*: Arterial therapeutic procedures (excluding aortic), Vascular imaging procedures NEC, Vascular therapeutic procedures NEC; *PT:* Transcatheter arterial chemoembolization.

[d]Includes the MedDRA *HLGT*: Vascular hypertension disorders; *PT*: BP abnormal, BP ambulatory abnormal, BP ambulatory increased, BP diastolic abnormal, BP diastolic increased, BP increased, BP systolic abnormal, BP systolic increased.

[e]Includes the MedDRA *HLGT:* Nephropathies; *HLT:* Renal failure and impairment; *PT:* Blood creatinine abnormal, Blood creatinine increased, Creatinine renal clearance abnormal, Creatinine renal clearance decreased, Glomerular filtration rate abnormal, Glomerular filtration rate decreased, Albuminuria, Proteinuria.

[f]Includes the MedDRA *HLT:* Diabetes mellitus (including subtypes), Hyperglycemic conditions NEC; *PT:* Blood glucose increased, Glycosylated hemoglobin increased.

[g]Includes the MedDRA *PT:* Hyperuricemia, Blood uric acid increased.

[h]Includes the MedDRA *HLGT*: Heart failures.

Please note that this summary only contains information from the full scientific article:

View Scientific Article

Plain Language Summary

Comparing bosutinib with imatinib in people with newly diagnosed chronic myeloid leukemia: 5-year follow-up results

Bosutinib <boh-SOO-tih-nib>

Chromosome <KROH-muh-some>

Chronic myeloid leukemia <KRAH-nik MY-eh-loyd loo-KEE-mee-uh>

Imatinib <ih-MA-tih-nib>

Tyrosine kinase inhibitor <TY-ruh-seen KY-nays in-HIH-bih-ter>

Date of summary: April 2022

Study number: NCT02130557
Study start date: July 2014
Study end date: April 2020

The full title of this article: Bosutinib versus imatinib for newly diagnosed chronic phase chronic myeloid leukemia: final results from the BFORE trial

Key takeaway

- In this study, people who took Bosulif (bosutinib) for treating newly diagnosed chronic myeloid leukemia (CML for short) had a quicker and greater response to treatment than those who took imatinib.
- Most of the side effects that patients experienced could be controlled with help from the doctors, and no new or unexpected side effects were seen with longer follow-up.

The purpose of this plain language summary is to help you to understand the findings from recent research.

- Bosutinib and imatinib are approved to treat the condition under study that is discussed in this summary.
- This summary reports the results of a single study. The results of this study may differ from those of other studies. Health professionals should make treatment decisions based on all available evidence, not on the results of a single study.

More information can be found in the scientific article of this study, which you can access here: View Scientific Article

Additional information

More information can be found in the scientific article of this study, which you can access here:

View Scientific Article

For more information on on this study, please visit:

https://www.clinicaltrials.gov/ct2/show/NCT02130557

For more information on clinical studies in general, please visit:

https://www.clinicaltrials.gov/ct2/about-studies/learn

http://www.cancerresearchuk.org/about-cancer/find-a-clinical-trial/what-clinical-trials-are

Bosutinib <boh-SOO-tih-nib>

Chromosome <KROH-muh-some>

Chronic myeloid leukemia <KRAH-nik MY-eh-loyd loo-KEE-mee-uh>

Imatinib <ih-MA-tih-nib>

Tyrosine kinase inhibitor <TY-ruh-seen KY-nays in-HIH-bih-ter>

What did this study look at?

- Chronic myeloid leukemia (CML for short) is a type of cancer that affects mainly white blood cells. Chronic means that it tends to progress slowly over many years.
  - CML is caused by formation of an abnormal gene called BCR-ABL1.
  - Genes are segments of DNA that tell cells in the body how to make a single protein. They are found in structures called chromosomes within each cell of the body.
  - The BCR-ABL1 gene is found in a chromosome called the Philadelphia chromosome and can be identified in CML cells and in some acute lymphoblastic leukemia cancer cells.
- Bosutinib is a type of medicine known as a tyrosine kinase inhibitor (TKI for short).
  - Tyrosine kinases are proteins in the body that control how cells grow and divide.
  - The BCR-ABL1 gene makes a tyrosine kinase that is more active than normal and helps leukemia cells grow.
  - Bosutinib works by blocking the more active tyrosine kinase in the cancer cells, causing them to die.
- In this study, researchers compared bosutinib with another TKI, imatinib, as a treatment for people with newly diagnosed CML.
- This summary describes the efficacy (efficacy is how well a drug works in a clinical trial) and safety of bosutinib compared to imatinib in this group of people after 5 years of follow-up.

Where is this study in the drug development timeline?

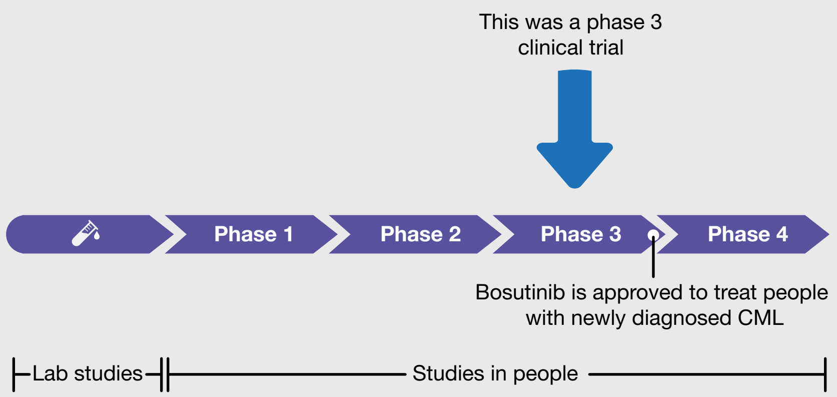

Additional information

More information can be found in the scientific article of this study, which you can access here:

View Scientific Article

For more information on on this study, please visit:

https://www.clinicaltrials.gov/ct2/show/NCT02130557

For more information on clinical studies in general, please visit:

https://www.clinicaltrials.gov/ct2/about-studies/learn

http://www.cancerresearchuk.org/about-cancer/find-a-clinical-trial/what-clinical-trials-are

Bosutinib <boh-SOO-tih-nib>

Chromosome <KROH-muh-some>

Chronic myeloid leukemia <KRAH-nik MY-eh-loyd loo-KEE-mee-uh>

Imatinib <ih-MA-tih-nib>

Tyrosine kinase inhibitor <TY-ruh-seen KY-nays in-HIH-bih-ter>

Who took part in this study?

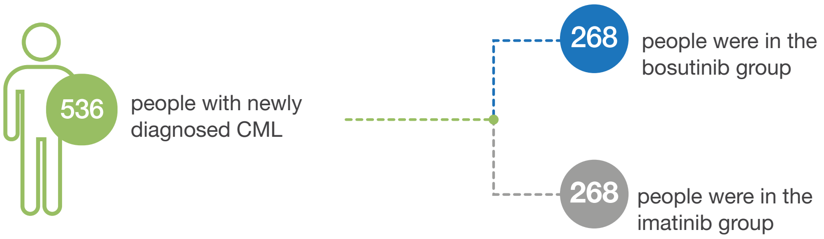

Although 268 people started the study in the imatinib group, 3 did not receive treatment.

- At the start of the study, researchers grouped people with CML based on the risk of their CML rapidly progressing and their likelihood of survival.
  - This grouping (known as the Sokal risk group) was based on various factors, including age, blood test results, and other clinical features.

What were the results of the study?

At the end of the study, in both treatment groups:

9 in 10 people were followed for the 5 years the study lasted

6 in 10 people were still receiving treatment at the end of the study

Half of the people took their medication for at least 55 months

Efficacy

- Researchers looked at whether treatment with bosutinib or imatinib lowered the amount of the BCR-ABL1 gene in the blood.
  - People who respond well to treatment have lower BCR-ABL1 levels. This means they have fewer leukemia cells containing the BCR-ABL1 gene.
- People who took bosutinib had lower levels of the BCR-ABL1 gene compared to those who took imatinib.

During the 5-year period people had:	Of the people who took bosutinib	Of the people who took imatinib
**Low** levels of the BCR-ABL1 gene (also known as a major molecular response or MMR for short) after taking their medication	74%	65%
**Very** low levels of the BCR-ABL1 gene (also known as molecular response 4 or MR^4 for short) after taking their medication	58%	48%
**Extremely** low levels of the BCR-ABL1 gene (also known as molecular response 4.5 or $MR^{4.5}$ for short) after taking their medication	47%	37%

Additional information

More information can be found in the scientific article of this study, which you can access here:

View Scientific Article

For more information on on this study, please visit:

https://www.clinicaltrials.gov/ct2/show/NCT02130557

For more information on clinical studies in general, please visit:

https://www.clinicaltrials.gov/ct2/about-studies/learn

http://www.cancerresearchuk.org/about-cancer/find-a-clinical-trial/what-clinical-trials-are

Bosutinib <boh-SOO-tih-nib>

Chromosome <KROH-muh-some>

Chronic myeloid leukemia <KRAH-nik MY-eh-loyd loo-KEE-mee-uh>

Imatinib <ih-MA-tih-nib>

Tyrosine kinase inhibitor <TY-ruh-seen KY-nays in-HIH-bih-ter>

- When comparing BCR-ABL1 gene levels in people taking bosutinib and those taking imatinib, the largest difference was seen among people in the Sokal high-risk group.

The proportion of people with low levels of the BCR-ABL1 gene (known as MMR), by risk group

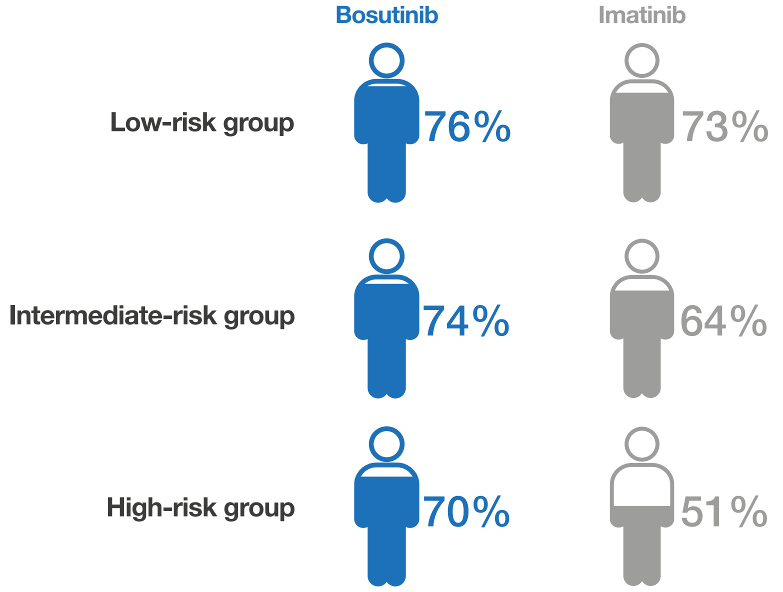

- In people who had lower levels of the BCR-ABL1 gene after treatment:
  - low BCR-ABL1 gene levels were achieved more quickly with bosutinib than in people taking imatinib
  - low levels of the BCR-ABL1 gene were maintained for a similar length of time with either treatment.
- There were no differences between treatments in:
  - how long people lived overall
  - how long people lived without their CML returning.

The likelihood that a person is alive after 5 years

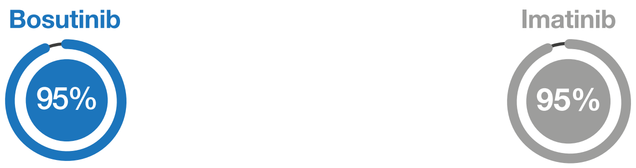

The likelihood that after 5 years, a person's CML returns or they die

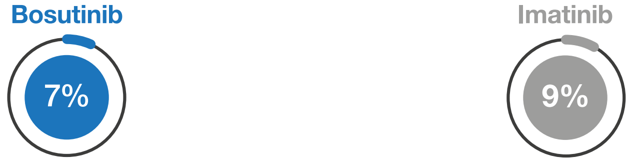

Additional information

More information can be found in the scientific article of this study, which you can access here:

View Scientific Article

For more information on on this study, please visit:

https://www.clinicaltrials.gov/ct2/show/NCT02130557

For more information on clinical studies in general, please visit:

https://www.clinicaltrials.gov/ct2/about-studies/learn

http://www.cancerresearchuk.org/about-cancer/find-a-clinical-trial/what-clinical-trials-are

Bosutinib <boh-SOO-tih-nib>

Chromosome <KROH-muh-some>

Chronic myeloid leukemia <KRAH-nik MY-eh-loyd loo-KEE-mee-uh>

Imatinib <ih-MA-tih-nib>

Tyrosine kinase inhibitor <TY-ruh-seen KY-nays in-HIH-bih-ter>

Safety

- Most people who took part in this study experienced side effects, with most side effects occurring during the first year of treatment.
  - A side effect is something (expected or unexpected) that you feel was caused by a medicine or treatment you take.

The proportion of people who had side effects

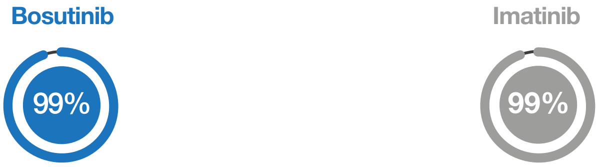

The proportion of people who had severe* side effects

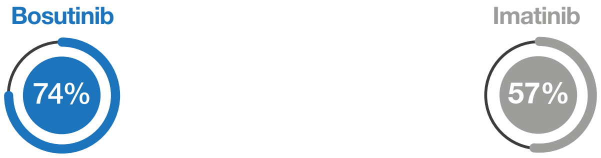

* A side effect is considered 'severe' when it limits daily activities such as bathing and dressing, is disabling or is medically significant, or could be life-threatening, need hospital care, or cause lasting problems.

- Researchers looked at the type of side effects people had while taking their medicine. Compared to people who took imatinib, those who took bosutinib had:
  - more side effects that affected their liver, skin, or stomach and intestines
  - less swelling in parts of their body, and less pain in their muscles and bones.

Additional information

More information can be found in the scientific article of this study, which you can access here:

View Scientific Article

For more information on on this study, please visit:

https://www.clinicaltrials.gov/ct2/show/NCT02130557

For more information on clinical studies in general, please visit:

https://www.clinicaltrials.gov/ct2/about-studies/learn

http://www.cancerresearchuk.org/about-cancer/find-a-clinical-trial/what-clinical-trials-are

Bosutinib <boh-SOO-tih-nib>

Chromosome <KROH-muh-some>

Chronic myeloid leukemia <KRAH-nik MY-eh-loyd loo-KEE-mee-uh>

Imatinib <ih-MA-tih-nib>

Tyrosine kinase inhibitor <TY-ruh-seen KY-nays in-HIH-bih-ter>

Additional information

More information can be found in the scientific article of this study, which you can access here:

View Scientific Article

For more information on on this study, please visit:

https://www.clinicaltrials.gov/ct2/show/NCT02130557

For more information on clinical studies in general, please visit:

https://www.clinicaltrials.gov/ct2/about-studies/learn

http://www.cancerresearchuk.org/about-cancer/find-a-clinical-trial/what-clinical-trials-are

The proportion of people who had certain types* of side effects

	Bosutinib	Imatinib
Gastrointestinal side effects (such as diarrhea and feeling sick)	80%	62%
Low levels of some blood cells (such as low levels of platelets and neutrophils)	48%	47%
Liver side effects (such as increased liver enzymes)	44%	16%
Skin side effects (such as rashes and acne)	39%	26%
Pain in muscles and bones (such as joint and back pain)	35%	60%
Swelling in parts of the body (such as swelling of the hands or face)	16%	43%
High blood pressure	10%	11%
Kidney side effects (such as sudden kidney damage)	10%	10%
Heart side effects (such as an irregular heart rate)	10%	9%
Metabolic side effects (such as high cholesterol and high blood sugar)	9%	8%
Veins and artery side effects (such as chest pains and strokes)	8%	3%
An abnormal build-up of fluid around the lungs or the heart	6%	2%

* Using the Medical Dictionary for Regulatory Activities (MedDRA for short), similar types of side effects were grouped.

Bosutinib <boh-SOO-tih-nib>

Chromosome <KROH-muh-some>

Chronic myeloid leukemia <KRAH-nik MY-eh-loyd loo-KEE-mee-uh>

Imatinib <ih-MA-tih-nib>

Tyrosine kinase inhibitor <TY-ruh-seen KY-nays in-HIH-bih-ter>

- To help reduce side effects, researchers sometimes recommend that people:
  - reduce the dose of bosutinib or imatinib they take,
  - temporarily stop taking the medication, or
  - take another medication to treat the side effects (like an anti-diarrhea medication).

Proportion of people who had the dose of their medication reduced due to side effects

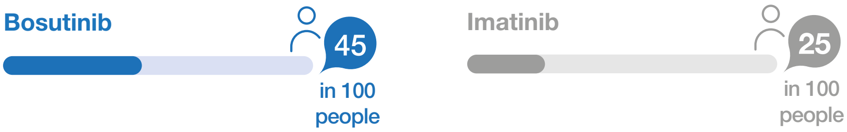

Proportion of people who temporarily stopped taking their medication due to side effects

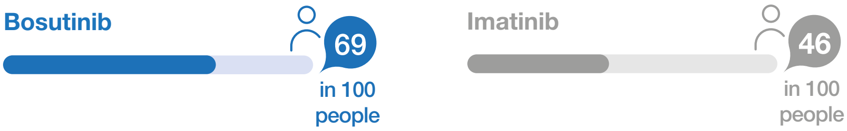

- The most common reasons for people completely stopping their treatment were side effects and the treatment not working well enough.

Proportion of people completely stopping treatment due to side effects

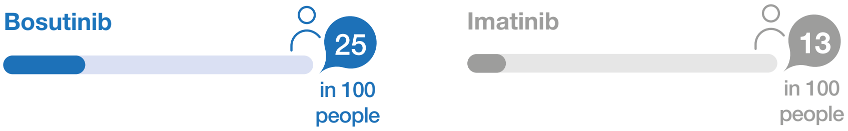

Proportion of people completely stopping treatment due to their treatment not working well enough

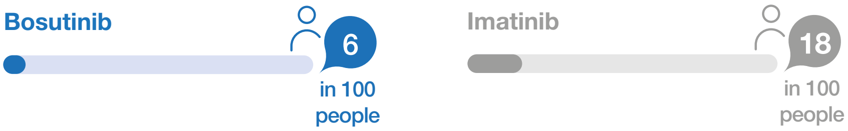

Additional information

More information can be found in the scientific article of this study, which you can access here:

View Scientific Article

For more information on on this study, please visit:

https://www.clinicaltrials.gov/ct2/show/NCT02130557

For more information on clinical studies in general, please visit:

https://www.clinicaltrials.gov/ct2/about-studies/learn

http://www.cancerresearchuk.org/about-cancer/find-a-clinical-trial/what-clinical-trials-are

Bosutinib <boh-SOO-tih-nib>

Chromosome <KROH-muh-some>

Chronic myeloid leukemia <KRAH-nik MY-eh-loyd loo-KEE-mee-uh>

Imatinib <ih-MA-tih-nib>

Tyrosine kinase inhibitor <TY-ruh-seen KY-nays in-HIH-bih-ter>

Additional information

More information can be found in the scientific article of this study, which you can access here:

View Scientific Article

For more information on on this study, please visit:

https://www.clinicaltrials.gov/ct2/show/NCT02130557

For more information on clinical studies in general, please visit:

https://www.clinicaltrials.gov/ct2/about-studies/learn

http://www.cancerresearchuk.org/about-cancer/find-a-clinical-trial/what-clinical-trials-are

What were the main conclusions reported by the researchers?

- After 5 years, compared to imatinib, people who took bosutinib:
  - had an earlier response and lower BCR-ABL1 gene levels
  - lived for a similar length of time overall
  - lived for a similar length of time without their CML returning or dying.
- Most of the side effects that patients experienced could be controlled with help from the doctors, and no new or unexpected side effects were seen with longer follow-up.
- These results support the use of bosutinib as a treatment for people with newly diagnosed CML.

Who sponsored this study?

Pfizer Inc.
235 East 42nd Street, NY, NY 10017
Phone (United States): +1 212-733-2323

Pfizer would like to thank all of the people who took part in this study.

Scientific Article

Brümmendorf TH, Cortes JE, Milojkovic D, et al. Bosutinib versus imatinib for newly diagnosed chronic phase chronic myeloid leukemia: final results from the BFORE trial. Leukemia, 2022; doi: 10.1038/s41375-022-01589-y

Acknowledgements

Writing support for this summary was provided by Gemma Shay, PhD, and David Sunter, PhD, CMPP, of Engage Scientific Solutions, and was funded by Pfizer. The original authors of the full article were involved in preparing this summary.
